# Diversity of bacterial communities on the facial skin of different age-group Thai males

**DOI:** 10.7717/peerj.4084

**Published:** 2017-11-21

**Authors:** Alisa Wilantho, Pamornya Deekaew, Chutika Srisuttiyakorn, Sissades Tongsima, Naraporn Somboonna

**Affiliations:** 1 Genome Technology Research Unit, National Center for Genetic Engineering and Biotechnology, Pathum Thani, Thailand; 2 Department of Microbiology, Faculty of Science, Chulalongkorn University, Bangkok, Thailand; 3 Division of Dermatology, Department of Medicine, Phramongkutklao Hospital, Bangkok, Thailand

**Keywords:** Human microbiome, Bacteria diversity, Skin, 16S rRNA gene, Next generation sequencing, Aging

## Abstract

**Background:**

Skin microbiome varies from person to person due to a combination of various factors, including age, biogeography, sex, cosmetics and genetics. Many skin disorders appear to be related to the resident microflora, yet databases of facial skin microbiome of many biogeographies, including Thai, are limited.

**Methods:**

Metagenomics derived B-RISA and 16S rRNA gene sequencing was utilized to identify the culture-independent bacterial diversity on Thai male faces (cheek and forehead areas). Skin samples were categorized (grouped) into (i) normal (*teenage.hea*) and (ii) acne-prone (*teenage.acn*) young adults, and normal (iii) middle-aged (*middle.hea*) and (iv) elderly (*elderly.hea*) adults.

**Results:**

The 16S rRNA gene sequencing was successful as the sequencing depth had an estimated >98% genus coverage of the true community. The major diversity was found between the young and elderly adults in both cheek and forehead areas, followed by that between normal and acne young adults. Detection of representative characteristics indicated that bacteria from the order Rhizobiales, genera *Sphingomonas* and *Pseudoalteromonas*, distinguished the *elderly.hea* microbiota, along the clinical features of wrinkles and pores. Prediction of the metabolic potential revealed reduced metabolic pathways involved in replication and repair, nucleotide metabolism and genetic translation in the *elderly.hea* compared with that in the *teenage.hea*. For young adults, some unique compositions such as abundance of *Propionibacterium acnes* and *Staphylococcus epidermidis*, with a minor diversity between normal and acne skins, were detected. The metabolic potentials of the acne vs. normal young adults showed that *teenage.acn* was low in many cellular processes (e.g., cell motility and environmental adaptation), but high in carbohydrate metabolism, which could support acne growth. Moreover, comparison with the age-matched males from the US (Boulder, Colorado) to gain insight into the diversity across national biogeography, revealed differences in the distribution pattern of species, although common bacteria were present in both biogeographical samples. Furthermore, B-RISA served as a crosscheck result to the 16S rRNA gene sequencing (i.e., differences between teenage and elderly microbiota).

**Conclusions:**

This study revealed and compared the microbial diversity on different aged Thai male faces, and included analyses for representing the bacterial flora, the clinical skin characteristics, and comparison with the US age-matched. The results represent the first skin microbiota of Thai males, and helps the design of a large-scale skin microbiome study of Thais. The findings of the diversity among ages, skin type and national biogeography supported the importance of these traits in the skin microbiome and in developing a safe and sustainable treatment for acne and aging skin diseases.

## Introduction

Following advances in metagenomics and next generation sequencing (NGS), the Human Microbiome Project (HMP) was established and several aspects of human microbial symbionts have been deciphered (HMP, 2012; Cosseau et al., 2016). Different organs and parts of the human body harbor an array of microbiota according to specific niche characteristics (Zhou et al., 2013). The anatomy and physiology are key factors in determining the skin bacterial diversity, such as the forehead, forearm, palm, finger, back of knee and foot (Costello et al., 2009; Zhou et al., 2013). Even on a particular niche of the body (e.g., face), the skin microbiota are still complicated by a combination of both internal and external factors, including but not limited to, age, sex, biogeography (climate and environment), genetics, cosmetics, diet, immune response, hormones and daily lifestyles (Cho & Blaser, 2012; HMP, 2012).

Generally, skin bacteria are dominated by members of the Actinobacteria, Proteobacteria, Firmicutes and Bacteroidetes phyla. Within these phyla, genera in *Propionibacterium*, *Corynebacterium*, *Staphylococcus*, *Micrococcus* and *Brevibacterium* are common (Van Rensburg et al., 2015; Mukherjee et al., 2016). The human skin bacteria are initiated since the different modes of delivery. For instances, *Lactobacillus* and *Prevotella* are predominant on the infant skin born naturally through the vagina, whereas *Acinetobacter* and *Staphylococcus* are predominant on those born by caesarian section (Dominguez-Bello et al., 2010). Bacterial diversity continues to vary through time, due in part to the internal and external combinatory factors of each individual (Capone et al., 2011; SanMiguel & Grice, 2015). Within the same age range, the skin type, such as moist and sebaceous skin vs. dry skin, also plays a role in the bacterial diversity (Grice et al., 2009). Mukherjee et al. (2016) analyzed the correlation between the sebum and hydration levels on the cheek and forehead microbiota of 30 Indian female volunteers, and found the sebum and hydration levels provided predictive power with respect to the nature and microbiota of forehead and cheek skins. This is consistent with earlier studies, which reported that the microbial compositions play roles in the “normal,” “predisposed” and “disease” states of the skin health, such as in atopic dermatitis and chronic skin ulcers (Costello et al., 2009; Cho & Blaser, 2012; Van Rensburg et al., 2015). It was hypothesized that a loss of protective bacteria led to the outgrowth of pathogenic bacteria (Van Rensburg et al., 2015; Barnard et al., 2016). Kong et al. (2012) reported that children with a severe level of atopic dermatitis had an increased level of *Staphylococcus aureus* but a decreased overall microbial diversity on the skin. Recently, based on 38 US acne patients and 30 US age matched controls, Barnard et al. (2016) found that the acne and health of the human facial skin was not associated with an abundant *Propionibacterium acnes* but with a disrupted microbiota. Accordingly, they proposed using probiotic and *P. acnes* phage as sustainable and safe strategies for maintaining skin health.

Possible reasons for the disrupted microbiota to cause acne can be many, such as diet habits (i.e., glycemic load and water intake), hours of quality sleep (i.e., affects emotional state), and genetics. This relationship is through the “gut-brain-skin theory” (Bowe, Patel & Logan, 2014). A healthy eating pyramid (types and proportions of food for a daily healthy eating), such as Mediterranean food that is rich in vegetable fibers, fruit antioxidants, vegetable and fish proteins, and low glycemic load, could change the gut microbiota (Wu et al., 2011; Queipo-Ortuno et al., 2012; Lopez-Legarrea et al., 2014). Clark, Haas & Sivamani (2017) reviewed the links between eating plant fibers and low glycemic food with altered gut microbiota and acne improvement. Another example was that the oral consumption of lactic acid bacteria has been shown to alleviate acne and mental stress (i.e., anxiety and depression). Lactic acid bacteria improve bowel function and reduce constipation, which is one underlying cause of acne and depression, and further, the diets and gut microbiota affect inflammatory signals in the human body (Bowe, Patel & Logan, 2014; Lopez-Legarrea et al., 2014). Other influence factors to acne include daily intake amount and types of beverages (e.g., milk vs. drinking water), and hours of sleep. Adebamowo et al. (2005) reported an association between teenage acne and milk (whole milk and skim milk) consumption, and Rigopoulos et al. (2007) reported that adequate hours of sleep could, in part, reduce teenage acne.

The present study utilized culture-independent metagenomics combined with bacterial-ribosomal intergenic spacer analysis (B-RISA) and 16S rRNA gene sequencing to disclose the bacterial communities of normal skin type (healthy face skin as inspected by dermatologist) Thai males of different age periods. These were categorized as young adults (*teenage.hea*), middle-aged (*middle.hea*) and elderly (*elderly.hea*) and, for young adults, the presence of mild-level acne vulgaris was also included (*teenage.acn*). Acne vulgaris, also called teenage acne, is a common skin disease among teenage and young adults. Approximately 80% of teenagers and young adults have or experienced this skin problem, because this age period is more prone to over active sebaceous glands, which cause plugged pores that promote the overgrowth of *Propionibacterium acnes* (James, 2005).

As for the methods, B-RISA served a pre-classification and cross-validation of the bacterial diversity without sequencing. B-RISA, denatured gradient gel electrophoresis (DGGE), and terminal restriction fragment length polymorphism (TRFLP) are established methods that classify bacterial diversity structures using electrophoresis technique (Fisher & Triplett, 1999; Danovaro et al., 2006; Sikaroodi & Gillevet, 2012; Gobet, Boetius & Ramette, 2014; Jami, Shterzer & Mizrahi, 2014). B-RISA relies on the characteristic DNA fragment lengths between the 16S and 23S rRNA genes upon bacterial species. Different species have different B-RISA lengths. Researches have compared the power of B-RISA and NGS in classifying bacterial community patterns, and reported the agreement in the term of the discriminating power among samples. Nevertheless, B-RISA yielded a much underestimated number of the species richness and provided no information regarding sequences or names of individual species (Grillevet, Sikaroodi & Torzilli, 2009; Sikaroodi & Gillevet, 2012; Gobet, Boetius & Ramette, 2014; Jami, Shterzer & Mizrahi, 2014; Dehingia et al., 2015). This study adopted B-RISA as an inexpensive and rapid bacterial community fingerprint tool prior to NGS.

For the 16S rRNA gene sequencing to obtain the bacterial taxonomic compositions, the study utilized mothur’s MiSeq standard operating procedures (SOP) (Schloss et al., 2009) and Metastats (White, Naharajan & Pop, 2009) to detect differentially abundant features, including representative bacterial species and clinical features (skin, diet and lifestyle), in the datasets. The study also compared the Thai male face skin microbiota with age-matched US biogeography (reference data from Costello et al., 2009), and the bacterial diversity and metabolic potentials between these two biogeographic regions were reported. Together, the skin microbiota variation according to age, skin features, lifestyles, and biogeography, and thus various aspects of the skin, will help to complete the skin microbiota knowledge and databases (Van Rensburg et al., 2015).

## Materials & Methods

### Sample collection

Study subjects were comprised of 40 healthy Thai male volunteers with no health disease and skin disorders, except for the presence of acne vulgaris, where stated. Subjects were classified as 10 normal face young adults (19–24 y; *teenage.hea*), 10 normal face middle-aged (32–38 y; *middle.hea*), 10 normal face elderly (51–57 y; *elderly.hea*) and 10 mild grade acne young adults (19–24 y; *teenage.acn*). Skin types were graded with classification support from a dermatologist (Table 1). Volunteers were recruited at Chulalongkorn University and Phramongkutklao Hospital, in Bangkok, during June–September 2013. The sample selection criteria and collection process followed the established procedures (Staudinger, Pipal & Redl, 2011; Ling et al., 2013). All samples were swabbed on the same sample areas of the face by the same investigator, and in the same swabbing manner to prevent possible variation across samples. Participants had used neither topical antibiotics in the past 7 d nor oral antibiotics in the past 3 d. Participants were refrained from washing their faces for approximately 8 h prior to sample collection. To minimize sample cross-contamination, subjects belonging to different skin groups were instructed to come for sample collection on a different week. For each individual, an area of 1 × 1 inch was swabbed on the forehead, as well as on both the right and left cheek. With the pooling of the two cheek swabs, this led to two clinical samples per volunteer: forehead and cheeks, and that two areas of the cheek (left and right) was taken into account in the estimates of DNA density per swab area. With 10 individuals per group and four groups in total, the study thereby contained 80 samples. Each skin swab sample was immediately stored in sterile saline-Tween solution (0.15 M NaCl and 0.1% (v/v) Tween 20) at −20 °C, and processed within 14 d. The study was approved by the Institute Review Board Royal Thai Army Medical Department Office, Phramongkutklao College of Medicine, Bangkok, Thailand (Q003h/56).

**Table 1:** Description of the facial skin characteristics from the four different subject groups.

Criteria	*teenage.hea*	*teenage.acn*	*middle.hea*	*elderly.hea*
Fitzpatrick skin type (I to VI)^a^	mostly IV, some V	mostly V, some IV	mostly V, some IV	mostly V, some IV
Roughness^b^	**0.20** ± 0.15	**0.48** ± 0.31	**0.38** ± 0.26	**0.43** ± 0.30
Wrinkles^b^	**0.10** ± 0.17	**0.30** ± 0.19	**0.33** ± 0.25	**0.60** ± 0.23
Mottled hyperpigmentation^b^	**0.03** ± 0.08	**0.08** ± 0.16	**0.25** ± 0.22	**0.23** ± 0.32
Flushing^b^	**0.23** ± 0.28	**0.23** ± 0.31	**0.10** ± 0.17	**0.13** ± 0.20
Elasticity^b^	**0.00** ± 0.00	**0.00** ± 0.00	**0.00** ± 0.00	**0.00** ± 0.00
Pore^b^	**0.25** ± 0.15	**0.50** ± 0.22	**0.53** ± 0.24	**0.55** ± 0.27
Acne^b^	**0.03** ± 0.07	**0.40** ± 0.26	**0.03** ± 0.10	**0.00** ± 0.00
Oil zone^b^	**0.40** ± 0.29	**0.70** ± 0.35	**0.30** ± 0.41	**0.00** ± 0.00
Healthy daily food pyramid	80%	90%	80%	90%
8–14 glasses of drinking water per day	70%	50%	90%	80%
8 h of sleep per day	30%	30%	40%	50%
Daily makeup	0%	0%	0%	0%
Sunlight activities	60%	40%	50%	50%

**Notes.**

a Fitzpatrick skin types I to VI are defined as follows: ‘I’, means always burns, never tans (pale white skin); ‘II’, means always burns easily, tans minimally (white skin); ‘III’, means burns moderately, tans uniformly (light brown skin); ‘IV’, means burns minimally, always tans well (moderate brown skin); ‘V’, means rarely burns, tans profusely (dark brown skin); and ‘VI’, means never burns (deeply pigmented dark brown to black skin).

b Degree of roughness, wrinkle, mottled hyperpigmentation, flushing, pore, acne, and oil zone range from 0 (none) to 1 (maximal degree); except elasticity that ranges from −1 (minimal degree) to 0 (normal). All data represent the **average** ± standard deviation (S.D.) from 10 subjects per group. Note healthy daily food pyramid intake and below questions the data were collected as ‘yes’ or ‘no’, hence the percentage was calculated and no S.D. is available.

### Total bacterial DNA extraction and DNA quality examination

Total bacterial DNA was extracted from each sampling using GF-1 Bacterial DNA Extraction Kit (Vivantis, Selangor Darul Ehsan, Malaysia), according to the manufacturer’s protocol. The quality and quantity of the extracted DNA were determined by NanoDrop spectrophotometry and agarose gel electrophoresis. This DNA was used for B-RISA and 16S rRNA MiSeq.

### B-RISA

The methods of B-RISA, including primers 16S-1392F and 23S-125R (Table S1) and thermocycling parameters (95 °C for 4 min, and 35 cycles of 94° 1 min, 55 °C 1 min and 72 °C 10 s, followed by 72 °C 2 min), followed established protocols (Fisher & Triplett, 1999; Hewson & Fuhrman, 2004; Danovaro et al., 2006). B-RISA targets an intergenic region between the 16S and 23S rRNA genes, where different bacteria specie (or group) is denoted the different size length based on electrophoresis technique. Thus, the community of bacteria are analyzed by the different size lengths composition (Gobet, Boetius & Ramette, 2014; Jami, Shterzer & Mizrahi, 2014; Dehingia et al., 2015). Each 20 µl B-RISA reaction comprised 1× EmeraldAmp^®^ GT PCR Master Mix (TaKaRa, Shiga, Japan), 0.3 µM of each primer, and 50 ng of the total bacterial DNA. Independent replicate reactions were performed per samples. The samples were analyzed for the different size lengths compositions on 1.75% agarose gel electrophoresis.

### 16S rRNA gene library preparation and MiSeq sequencing

Universal prokaryote primers 338F and 803R for 16S rRNA gene V3–V4 with the appended eight-nucleotide barcode on the 5′ tail of the oligo sequences were listed in Table S1 (Meyer, Stenzel & Hofreiter, 2008; Somboonna et al., 2014; Somboonna et al., 2017). The PCR recipe and thermocycling parameters followed established protocols: 25-µl PCR reaction comprised 1× EmeraldAmp^®^ GT PCR Master Mix (TaKaRa, Shiga, Japan), 0.3 µM of each primer, and 50–100 ng of the total bacteria DNA; and the PCR conditions were 95 °C for 4 min, and 30 cycles of 94°C for 45 s, 50 °C for 55 s and 72 °C for 1 min 30 s, followed by 72 °C for 10 min (Meyer, Stenzel & Hofreiter, 2008; Somboonna et al., 2017). To prevent PCR stochastic bias, the template quantity and quality was adequate, and a minimum of three independent PCR reactions were performed per sample (Kennedy et al., 2014). The negative controls included a separate mock swabbing sample and no template control in the PCR, both of which showed no amplification. The 16S rDNA amplicons (∼466 bp) were purified using PureLink^®^ Quick Gel Extraction Kit (Invitrogen, New York, USA), quantified by Qubit (Life Technologies, Carlsbad, CA, USA), and determined a purity and size of the purified amplicon by Agilent 2100 Bioanalyzer (Agilent Technologies, Santa Clara, CA, USA). Due to the heterogeneity of some samples of the groups as was demonstrated by B-RISA banding (Table 2), DNA from same-group subjects that shared similar B-RISA profiles were pooled together in equal amplicon amount generating a total of 11 subgroups for Illumina MiSeq sequencing. MiSeq sequencing adaptors were added to each of the 11 subgroups using Nextara Index Kit (Illumina, San Diego, CA, USA), and purified using Agencourt AMPure Technology (Beckman Coulter, Brea, CA, USA). The products were quantified and size checked by Qubit (Life Technologies) and Agilent 2100 Bioanalyzer (Agilent Technologies), respectively. The libraries were also quantified by quantitative PCR using Kapa Biosystems Library Quantification Kit (Kapa Biosystems, Woburn, MA, USA). Paired-end sequencing, 2 × 250, was performed using the Illumina MiSeq platform following the manufacturer’s protocols at Chulalongkorn Medical Research Center (Bangkok, Thailand).

**Table 2:** Overview of the microbial communities, classified into 11 subgroups by B-RISA analysis. Relative abundance is the number of clinical samples out of 10 total per group that show particular B-RISA pattern. Different band size in B-RISA pattern denotes different bacteria species or groups; hence clinical samples with different bacteria community compositions were classified by different B-RISA banding patterns. Note the reported average bacterial DNA, in ng/ul and ng/swab area, may contain some human DNA.

Marker ∖ Groups	Healthy face teenagers			Acne face teenagers				Healthy face middle-ages			Health face elder-ages	
	cheeks	foreheads		cheeks		foreheads		cheeks	foreheads		cheeks	foreheads
Average bacteria DNA (ng/ μl)	0.77	1.33		0.73		1.21		3.54	1.59		0.9	4.04
Average bacteria DNA (ng/swab area, 1 × 1 in.^2^ left & right cheeks)	38.5	133		36.5		121		177	159		45	404
B-RISA patterns	1	2		2		2		No amplification	2		1	1
2,000 bp												
1,900 bp												
1,800 bp												
1,700 bp												
1,600 bp												
1,500 bp												
1,400 bp					(14.4%, 1,400 bp) –		(37.5%, 1,400 bp) –					
1,300 bp												
1,200 bp										(7.8%, 1,250 bp) –	(33.3%, 1,250 bp) –	(44.5%, 1,250 bp) –
1,100 bp				(30.5%, 1,100 bp) –	(21.6%, 1,100 bp) –	(42.8%, 1,100 bp) –			(14.2%, 1,150 bp) – (12.6%, 1,100 bp) –	(7.8%, 1,150 bp) –	(66.7%, 1,100 bp) –	
1,000 bp												(55.5%, 1,000 bp) –
900 bp	(20.3%, 950 bp) –	(27.2%, 950 bp) –		(40.7%, 950 bp) –	(14.4%, 950 bp) –		(41.7%, 950 bp) –		(24.6%, 950 bp) –	(19.4%, 950 bp) –		
800 bp	(21.1%, 800 bp) –	(33.2%, 800 bp) –		(28.8%, 800 bp) –	(25.6%, 800 bp) –	(40.6%, 800 bp) –	(12.5%, 800 bp) –		(29.6%, 800 bp) –	(33.3%, 800 bp) –		
700 bp									(18.9%, 700 bp) –	(11.6%, 700 bp) –		
600 bp	(17.2%, 650 bp) –	(39.6%, 650 bp) –	(33.3%, 600 bp) –									
500 bp	(24.9%, 580 bp) – (16.5%, 500 bp) –		(66.6%, 500 bp) –		(24%, 500 bp) –	(16.6%, 500 bp) –	(8.3%, 500 bp) –			(20.2%, 580 bp) –		
400 bp												
300 bp												
200 bp												
100 bp												
Relative abundance (out of 10 subjects)	10	8	2	7	3	9	1	10	5	5	9	9

### Availability of DNA sequencing reads

All nucleic acid sequences in this study were deposited at the NCBI Sequence Read Archive (SRA) database (accession number SRR3656927). The data pertaining to each stage of the following bioinformatic analyses were available at https://doi.org/10.6084/m9.figshare.5288338 (for pre-processing data, and scripts in the phylotype pipeline) and https://doi.org/10.6084/m9.figshare.5288332 (the results for OTU classification data).

### Bacterial composition and metabolic potential analyses

The raw sequences were categorized into groups based on the 5′ barcode sequences. The sequences were processed following mothur’s MiSeq SOP (Schloss et al., 2009; Kanehisa et al., 2016). The pre-processing steps included removal of (i) short read lengths of ≤100 nucleotides (excluding the primer and adaptor sequences), (ii) long homopolymers of ≥eight nucleotides, (iii) ambiguous nucleotides and (iv) chimera. The clean sequences were classified to the operational taxonomic unit (OTU) using the Ribosomal Database Project (RDP) Classifier (Wang et al., 2007) and Greengenes database (McDonald et al., 2012). A minimum bootstrap confidence score of 80% was used as a cutoff for taxonomic assignment. Genus and specie of OTU (GLOTU and SLOTU) followed the phylotype-based methods (Schloss & Handelsman, 2004). Alpha diversity by Shannon and Chao bacterial community richness, and the Good’s coverage index to estimate the data coverage of a community, were computed using mothur (Claesson et al., 2009; Schloss et al., 2009). Data normalization was performed to normalize the varying sequencing depth among 11 subgroups, using the mothur software (Schloss et al., 2009). For the groups with two B-RISA subgroups (Table 2: e.g., teenage.acn.foreheads1 and teenage.acn.foreheads2), the Student’s *t*-test (two-sided) and Boxplot variance analyses for these pair subgroups were computed. The data were merged if *p* > 0.05 (Table 3: i.e., teenage.acn.foreheads). The relative abundance of bacterial genera was visualized as Heatmap using the R statistics package. Additional mothur analyses included Venn diagram, and the community dissimilarity matrices by Morisita-Horn dissimilarity index (Schloss et al., 2009). The average-linkage dendrogram, used to cluster the community relationship among subgroups, was computed from the Morisita-Horn dissimilarity indices using the R statistics package. Non-metric multidimensional scaling (NMDS) along with analysis of molecular variant (AMOVA) statistics were computed using mothur, and visualized by R. Differentially abundant (representative) GLOTU and clinical features were detected using function Metastats in mothur with default parameters (White, Naharajan & Pop, 2009). The microbial metabolic potentials were predicted using PICRUSt software (Langille et al., 2013), which estimates the community metabolic potentials from the 16S rDNA sequencing data using the KEGG (Kyoto Encyclopedia of Genes and Genomes) database (Kanehisa et al., 2004; Kanehisa et al., 2016). Comparison of metabolic potentials between a pair of groups was computed in PICRUSt (Langille et al., 2013) based on a two-sided Welch’s *t*-test with adjusted Storey-Tibshirani’s (Storey & Tibshirani, 2003) and Benjamini–Hochberg’s (Benjamini & Hochberg, 1995) false discovery rates. Significant difference was accepted when the *q*-value was <0.05.

**Table 3:** Diversity estimators of the 16S rRNA sequencing communities at the GLOTU, FLOTU and OLOTU levels.

	OTUs	Chao	Shannon	Good’s coverage
**Groups∖ Genus level**				
elderly.hea.cheeks	167	263.67	3.0847	98.39
elderly.hea.foreheads	147	227.47	2.7066	98.52
middle.hea.foreheads1	109	193.56	2.5080	98.87
middle.hea.foreheads2	111	186.99	2.5515	98.83
teenage.acn.cheeks1	163	260.24	3.0019	98.28
teenage.acn.cheeks2	142	224.69	2.6665	98.52
teenage.acn.foreheads	136	236.68	2.5917	98.46
teenage.hea.cheeks	131	212.90	2.7649	98.68
teenage.hea.foreheads1	143	229.48	3.0622	98.58
teenage.hea.foreheads2	134	200.10	3.0875	98.65
**Groups∖ Family level**				
elderly.hea.cheeks	99	131.67	2.8307	99.29
elderly.hea.foreheads	93	135.94	2.5212	99.17
middle.hea.foreheads1	69	102.95	2.2783	99.41
middle.hea.foreheads2	76	111.55	2.4337	99.33
teenage.acn.cheeks1	101	136.84	2.7659	99.21
teenage.acn.cheeks2	95	130.59	2.4995	99.24
teenage.acn.foreheads	89	130.71	2.4378	99.19
teenage.hea.cheeks	81	114.74	2.5648	99.34
teenage.hea.foreheads1	90	128.98	2.8708	99.23
teenage.hea.foreheads2	87	110.30	2.7606	99.28
**Groups∖ Order level**				
elderly.hea.cheeks	47	59.39	2.2825	99.72
elderly.hea.foreheads	45	61.94	2.1036	99.66
middle.hea.foreheads1	34	48.39	1.9063	99.74
middle.hea.foreheads2	38	52.35	1.9449	99.72
teenage.acn.cheeks1	48	63.53	2.1330	99.67
teenage.acn.cheeks2	46	59.56	2.0276	99.72
teenage.acn.foreheads	44	59.26	2.0340	99.69
teenage.hea.cheeks	41	53.71	1.9518	99.72
teenage.hea.foreheads1	48	63.00	2.2126	99.67
teenage.hea.foreheads2	41	50.98	1.9927	99.72

For the Thai vs. US biogeography microbiota comparison, we compared the data with the culture-independent bacterial diversity databases of 6 normal age-matched male forehead-skin resided in Boulder, Colorado, USA (Costello et al., 2009): 5 males aged 30–35 y to compare with our *middle.hea* and 1 male aged 60 y to compare with our *elderly.hea*. The methods of sample collection and library preparation were similar: used 16S rRNA amplicon sequencing, except that Costello et al. (2009) did not require hours between bathe and sampling (ranged from 0.5 to several hours) vs. we sampled at ∼8 h (similar to Staudinger, Pipal & Redl, 2011; Ling et al., 2013, to allow establishment of natural flora on the volunteer faces), amplified the V2 region, which might overestimate species richness while V3–V4 region generally provides species richness comparable to the nearly full-length 16S rRNA gene (Youssef et al., 2009), and utilized 454 FLX sequencer. The Costello et al. (2009) sequencing data were downloaded and processed bioinformatic analyses the same procedures as our data for parallel data comparison.

## Results

### Physical and descriptive skin-related features and bacterial DNA extraction

The physical and descriptive skin-related characteristics of individuals were recorded, and the average results for the *teenage.hea*, *teenage.acn*, *middle.hea* and *elderly.hea* groups were displayed in Table 1. Among *teenage.hea*, *middle.hea* and *elderly.hea*, an increased intensity of skin roughness, wrinkles, mottled hyperpigmentation and pores were recorded with age, whereas skin flushing and the oil zone decreased. The acne vulgaris of the *teenage.acn* group was of a mild level, covering 40 ± 26% of the entire face and none on the trunk (Table 1). To control for contamination, a separate mock swabbing of clean air inside the biosafety cabinet (Van Rensburg et al., 2015), was included and no PCR amplicon occurred. The diet and lifestyles that might affect acne were also recorded. Participants reported generally healthy eating pyramids (80–90%) and no makeup use (Table 1).

Following bacterial DNA extraction of all 80 clinical forehead and cheeks samples, the low yield of bacterial DNA (Table 2: 0.73–4.04 ng/µl) indicated the light number of bacteria on the facial skin in nature (Costello et al., 2009; Zhou et al., 2013). The amount of extracted bacterial DNA was mostly greater from the forehead swabs than the cheeks, and from the elderly than the teenage skins (Table 2), where *teenage.hea* foreheads yielded 133 ng/swab area, *middle.hea* foreheads 159 ng/swab area and *elderly.hea* foreheads 404 ng/swab area. Some samples had an unsatisfactory DNA quality and so could not be successfully amplified and analyzed by B-RISA and by 16S rRNA gene. These were 10 samples from *middle.hea* cheeks, due to an average A_260_/A_280_ ratio of 1.10, and one sample each from *elderly.hea* cheeks and forehead, leaving nine out of 10 samples for this group (Table 2).

### Intragroup correlation and intergroup variation of bacterial communities by B-RISA

Of individual cheeks and foreheads from the same group showed a degree of relatedness in the B-RISA electrophoresis results, with uniform or just two banding patterns observed among the corresponding samples of cheek and forehead subgroup. This supported bacterial community similarity among intragroup samples, and led to 11 B-RISA classified subgroups (Table 2). The B-RISA results suggested the different bacterial community structures among the four groups (*teenage.hea*, *teenage.acn*, *middle.hea* and *elderly.hea*), and that the community structure of the *teenage.acn* foreheads were somewhat similar between the *teenage.hea* and *middle.hea* groups (Table 2).

### Comparative taxonomic profiles by 16S rRNA gene sequencing

**Figure 1:**
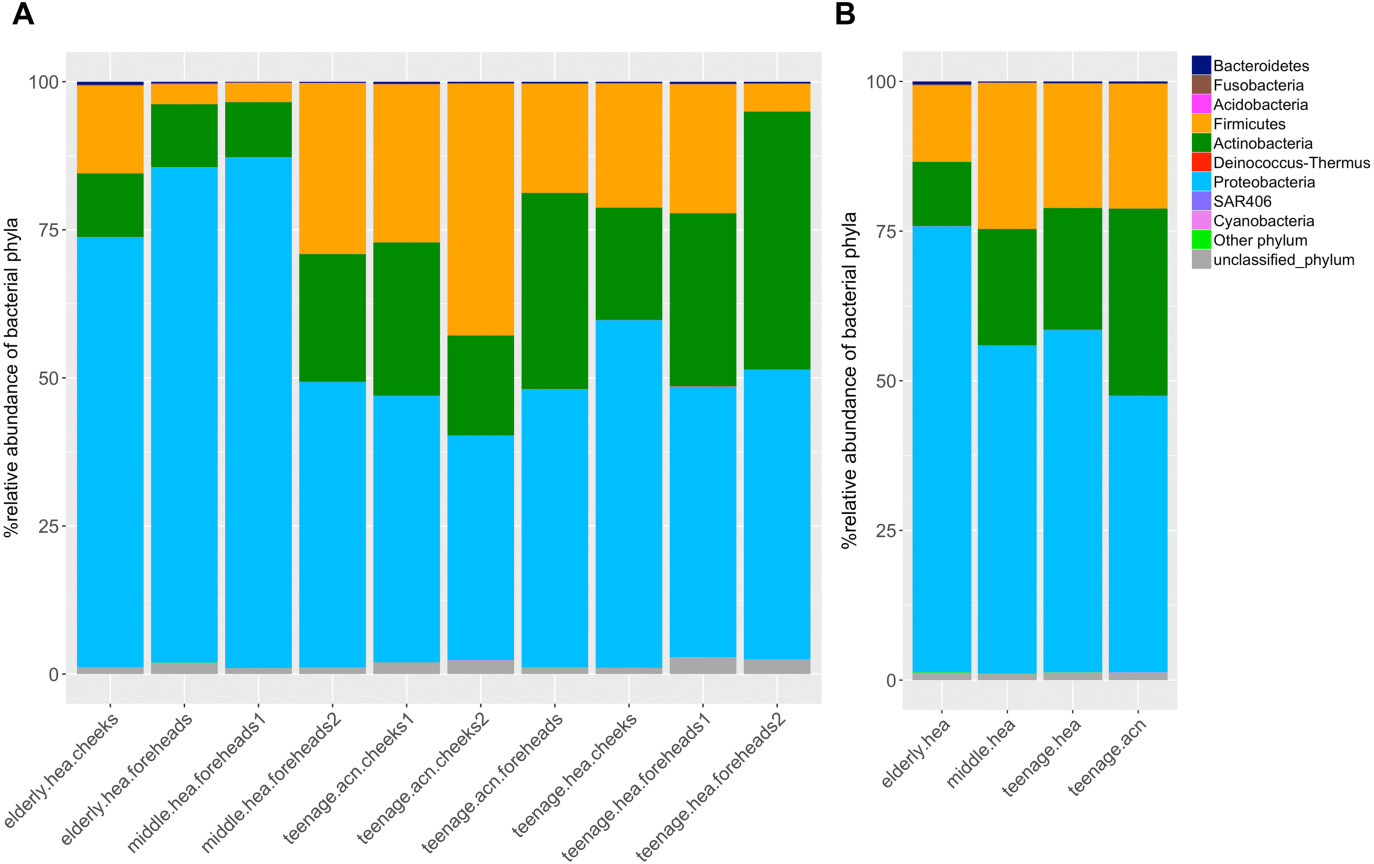
Diversity and relative abundances of bacterial phyla among (A) the 10 subgroups and (B) the four merged groups.

The 16S rRNA gene hypervariable V3–V4 sequencing was successful, as the sequencing depth of all 11 subgroups were sufficient to satisfy the >98% Good’s coverage index, and the taxonomic compositions could be annotated (Table 3). The V3–V4 region is accepted as most capable of bacterial species identification and hence of worldwide use (Youssef et al., 2009; Kennedy et al., 2014). Of groups that the B-RISA results suggested each consisted 2 subgroups (Table 2: *teenage.hea* foreheads, *teenage.acn* cheeks and foreheads, and *middle.hea* foreheads), the Student’s *t*-test (two-sided) and Boxplot variance of the 16S rDNA microbiota between the corresponding pairs were analyzed and the p statistics supported the community diversity in the *teenage.hea* foreheads1 and foreheads2, *teenage.acn* cheeks1 and cheeks2, and *middle.hea* foreheads1 and foreheads2 were significantly different (*p* ≤ 0.05) and so were retained as separate subgroups, whereas the *teenage.acn* foreheads1 and foreheads2 were not significantly different (*p* = 0.17) and so were merged to the single “*teenage.acn*.foreheads” subgroup. For the *teenage.acn* foreheads1 and foreheads2, the Boxplot analysis showed the overlapped variance, supported the merging of the “*teenage.acn*.foreheads” (Fig. S1). This led to 10 separate subgroups in the following analyses (e.g., Table 3 and Fig. 1).

The alpha diversity at the genus, family and order hierarchical levels of OTUs (GLOTU, FLOTU and OLOTU, respectively) of the 10 subgroups, using Chao’s taxon richness and Shannon’s diversity evenness (combining taxon richness and evenness), and the estimated sequencing coverage index (Good’s coverage), were summarized in Table 3. The community was comprised of 109–167 GLOTU, 69–101 FLOTUs and 34–48 OLOTUs. The number of OTUs and sample diversity indices indicated the diversity was relatively high in the elderly. For example, elderly.hea.cheeks was ranked in the top two highest numbers of OTUs at all three taxonomic levels, and had the highest Chao’s genus richness (Table 3). Moreover, estimating the genus richness from the rarefaction curve of the number of GLOTUs (*Y*-axis) against the number of random subsampling sequences (*X*-axis) suggested that for the cheeks the highest genus richness was found in the elderly.hea.cheeks and teenage.acn.cheeks1, and in the foreheads was found in the elderly.hea.foreheads and teenage.hea.foreheads1 (Figs. S2A and S2B). Together, the diversity indices highlighted the microbial diversity in the elderly and teenage groups.

### Intragroup correlation and intergroup variation of bacterial communities by 16S rRNA gene sequencing

**Figure 2:**
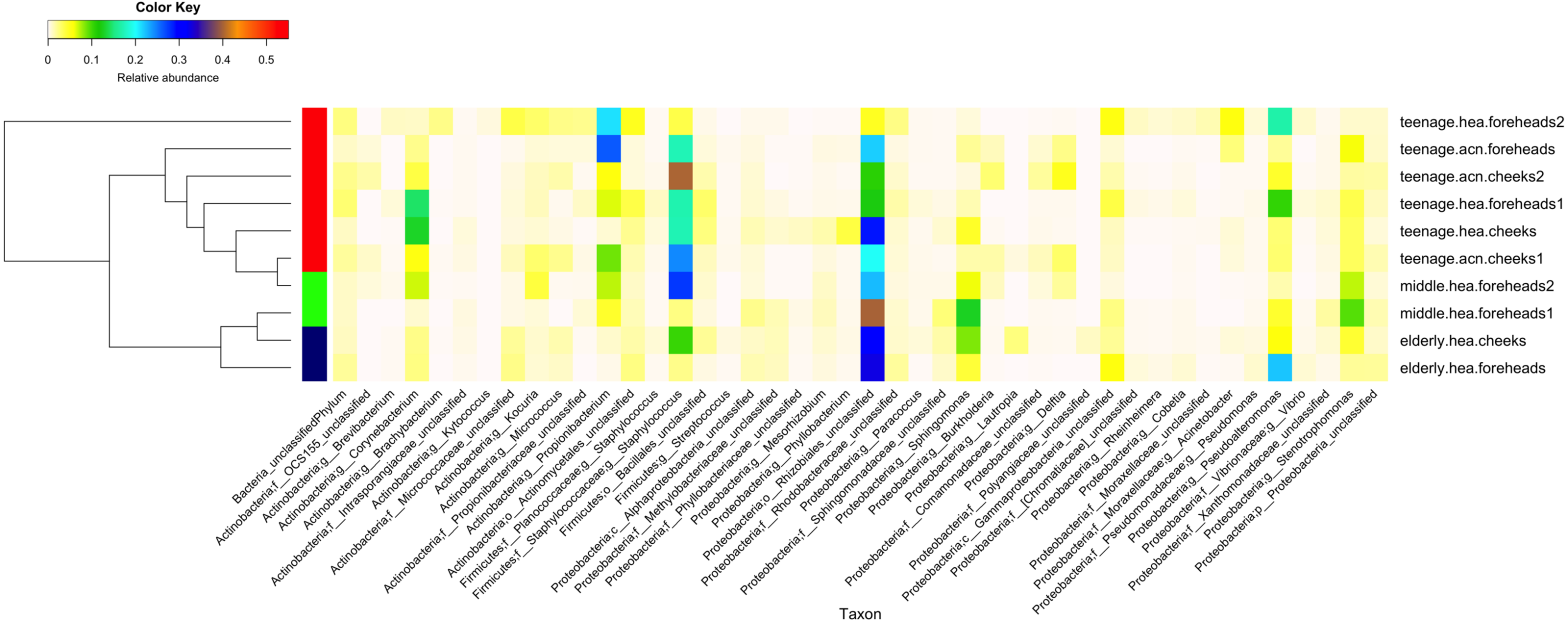
Diversity and relative abundances of bacterial community compositions (as GLOTU) among the 10 subgroups. Subgroups are clustered by dendrogram computed with Morisita-Horn dissimilarity indices: teenage (red), middle-aged (green) and elderly (navy). Bacterial genera of <0.05% relative abundance are not displayed.

The dominant phyla across the skin groups were Proteobacteria (37.9 to 86.2%) and Firmicutes (3.3% to 42.5) (Fig. 1A). These phyla have all been reported previously on the human face skin (Grice et al., 2009). The unclassified phyla averaged <1.6% (Fig. 1). Figure 2 showed the community distributions at the GLOTU level, in order of their community relatedness (clustering dendrogram on the left). For the young adults, abundant genera included *Staphylococcus* in phylum Firmicutes (*elderly.hea* 9.5%, *middle.hea* 23.4%, *teenage.acn* 19.6% and *teenage.hea* 17.6%), *Propionibacterium* in phylum Actinobacteria (*elderly.hea* 0.9%, *middle.hea* 7.0%, *teenage.acn* 23.3% and *teenage.hea* 1.8%) and *Corynebacterium* in phylum Actinobacteria (*elderly.hea* 2.4%, *middle.hea* 3.8%, *teenage.acn* 3.9% and *teenage.hea* 9.4%). For the elderly adults the abundant genera were an unclassified genus in the order Rhizobiales in class Alphaproteobacteria, phylum Proteobacteria (*elderly.hea* subgroups comprised 32.1% in average, *middle.hea* 31.3%, *teenage.acn* 17.5% and *teenage.hea* 15.3%) and *Sphingomonas* in phylum Proteobacteria (*elderly.hea* 6.4%, *middle.hea* 9.4%, *teenage.acn* 1.3% and *teenage.hea* 2.24%). Other prevalent bacteria included *Pseudoalteromonas* in phylum Proteobacteria (*elderly.hea* 13.9%, *middle.hea* 3.0%, *teenage.acn* 3.1% and *teenage.hea* 10.0%) and *Stenotrophomonas* in phylum Proteobacteria (*elderly.hea* 3.2%, *middle.hea* 8.3%, *teenage.acn* 3.7% and *teenage.hea* 2.7%) (Fig. 2).

Furthermore, the 16S rDNA V3–V4 sequences could phylotype *Staphylococcus* and *Propionibacterium* to the species level. *Staphylococcus* was found to mainly belong *S. epidermidis* (*elderly.hea* 3.13%, *middle.hea* 17.4%, *teenage.acn* 14.4% and *teenage.hea* 8.58%) and an unclassified OTU species (*elderly.hea* 5.54%, *middle.hea* 5.80%, *teenage.acn* 4.36% and *teenage.hea* 8.29%). The remaining species (<1%), in decreasing order of abundance, were *S. saprophyticus, S. aureus, S. haemolyticus, S. lugdunensis, S. pettenkoferi, S. sciuri* and *S. succinus*. Analysis at the species level found community differences between *teenage.hea* and *teenage.acn*, that the abundance of *S. epidermidis* was about two-fold greater in *teenage.acn*, and the third most common *Staphylococcus* species after the remaining unclassified in the *teenage.acn* was *S. aureus*, whereas that in the *teenage.hea* was *S. saprophyticus*. For *Propionibacterium*, the main species was *P. acnes* in *teenage.acn* (*elderly.hea* 0.76%, *middle.hea* 6.62%, *teenage.acn* 22.5% and *teenage.hea* 1.53%), while the rest (<1%) were an unclassified species and *P. granulosum*.

**Figure 3:**
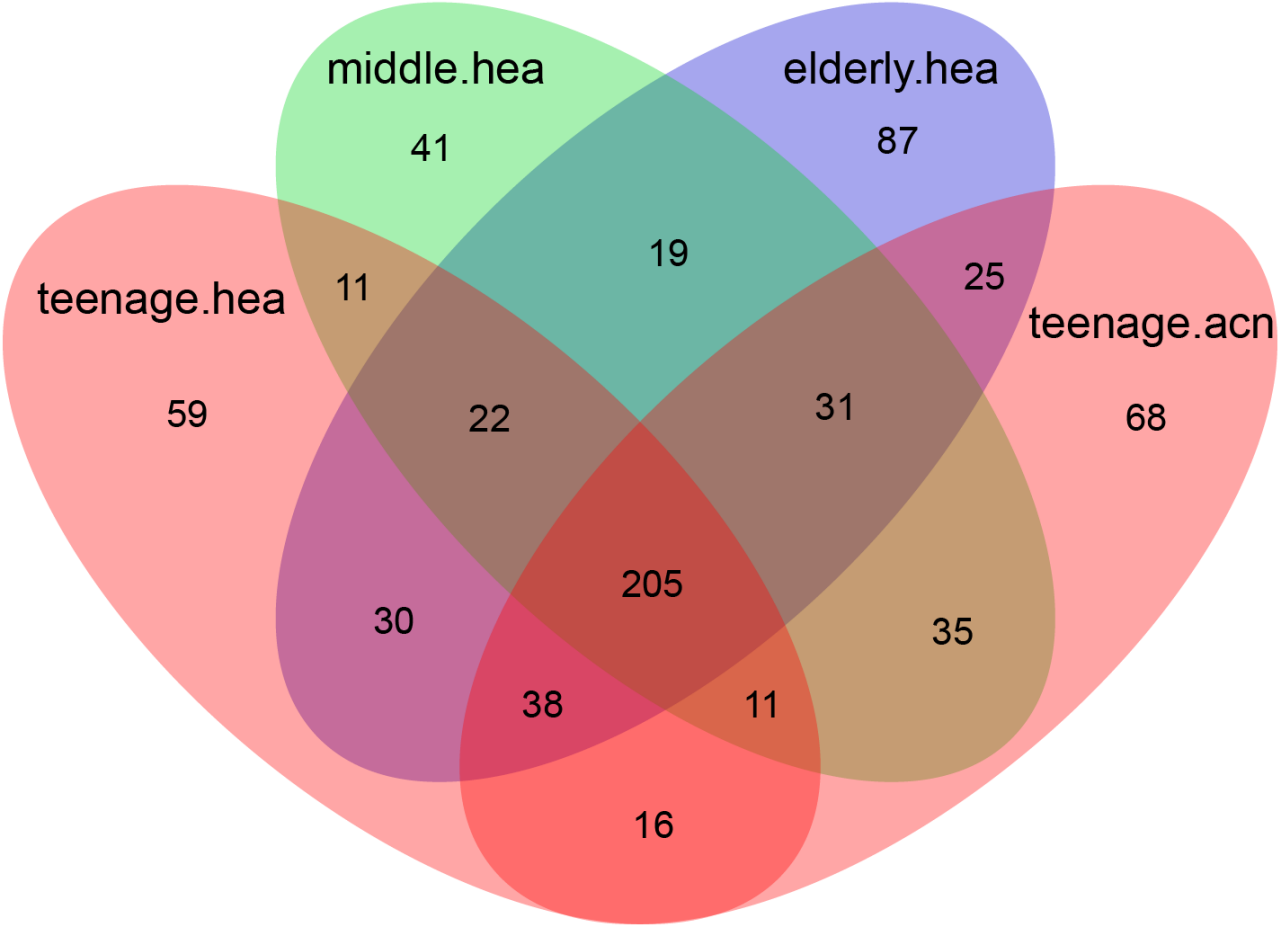
Venn diagram illustrating the overlapping GLOTUs among the four merged subject groups.

In summary, at the phylum level, Proteobacterium was prevalent in the elderly and middle aged, and Actinobacteria and Firmicutes in the young adults (both healthy and acne skins). At the genus level, Rhizobiales (unclassified genus) and *Sphingomonas* were prevalent in the elderly and middle-aged, *Pseudoalteromonas* in the elderly and normal young adults and *Stenotrophomonas* in the middle-aged and acne young adults. *Propionibacterium* (*P. acnes*) were prevalent in the acne young adults. *Staphylococcus* (*S. epidermidis, S. saprophyticus, S. aureus* and unclassified species) and *Corynebacterium* were prevalent in the middle-aged, and normal and acne young adults (Fig. 2). The similarity in the microbial population structures across groups caused the phylogenetic analysis (Fig. 2) to cluster cheeks and foreheads of the same corresponding groups (intragroup correlation), and young adults (in particular *teenage.hea*) separate from the elderly (intergroup variation).

**Figure 4:**
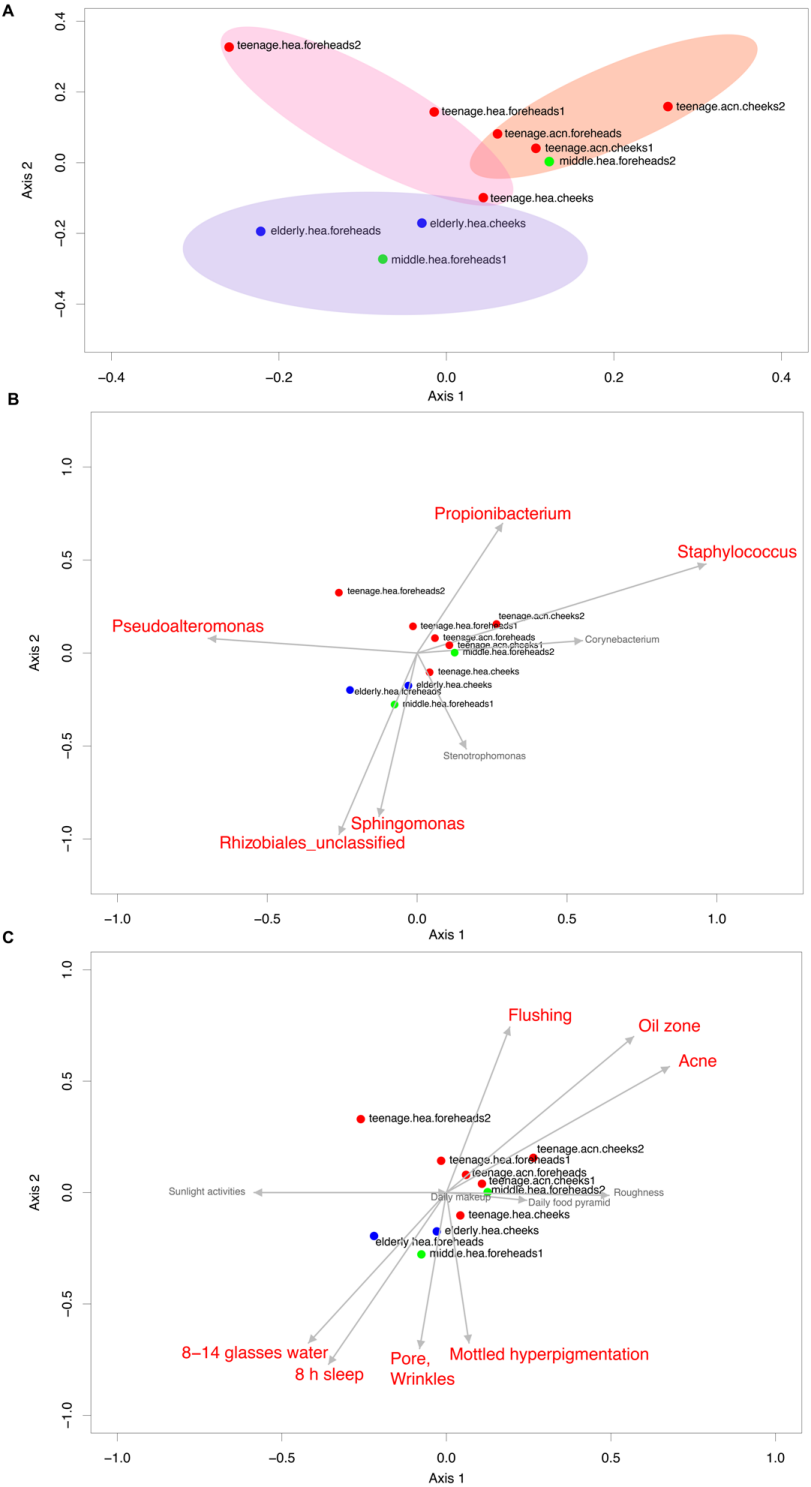
Non-metric multidimensional scaling based on Morisita-Horn dissimilarity indices, (A) without and (B, C) with Metastats analyses for representative (B) GLOTUs and (C) skin features. The vector length indicates the strength of the association. The direction infers the direction of the effect. In (A), circle with different filled color denotes community closeness (i.e., blue for *elderly.hea* and middle.hea.foreheads1, and red for *teenage.he* a and teenage.acn.foreheads). Circle size is proportional to the number of subjects. In (B) and (C), arrow with red font indicates the feature with significant statistics (*p* < 0.05), and arrow with the smaller size and in gray font (i.e., *Corynebacterium*, and roughness) indicates the feature with non-significant statistics (*p* > 0.05).

The Venn diagram displaying number of shared and unique GLOTUs across groups highlighted that *teenage.acn* shared a higher degree of GLOTU similarity with *middle.hea* than with *teenage.hea* (Fig. 3: *teenage.acn* & *middle.hea* 282 of 375 = 75.2%, *teenage.hea* & *middle.hea* 249 of 375 = 66.4%). This accounted for 65.7% (282 of 429) of the *teenage.acn* genus compositions. Furthermore, non-metric multidimensional scaling (NMDS) of the community correlation among subgroups against the two-dimensional distance located the *teenage.acn* communities nearby to the middle age (Fig. 4A).

The GLOTU compositions were tested by AMOVA to determine the statistical difference of their variation. No statistical significant intravariation between the forehead and cheeks of the same group was found, but significant intervariation was found between *elderly.hea* and *teenage.hea* (*p* = 0.019), and between *teenage.hea* and *teenage.acn* (*p* = 0.014) (Fig. 4A).

Metastats along AMOVA statistics was performed to identify any particular species of bacteria responsible for the direction of the community structure. The statistics suggested that the unclassified genus in the order Rhizobiales (*p* = 1e − 6) and *Sphingomonas* (8e−4) were distinguished to the *elderly.hea* community, and in an opposite direction to that driven by *Propionibacterium* (2.5e−2) and *Staphylococcus* (7e−6) for the young adult communities. *Pseudoalteromonas* (2.5e−2) drove a partial effect to the *elderly.hea* and *teenage.hea*, and a negative effect to the *teenage.acn* communities. *Corynebacterium* and *Stenotrophomonas* had no significant statistic impact on the skin community of middle-aged volunteers (9.8e−2 for *Corynebacterium* and 0.12 for *Stenotrophomonas*) (Fig. 4B).

**Figure 5:**
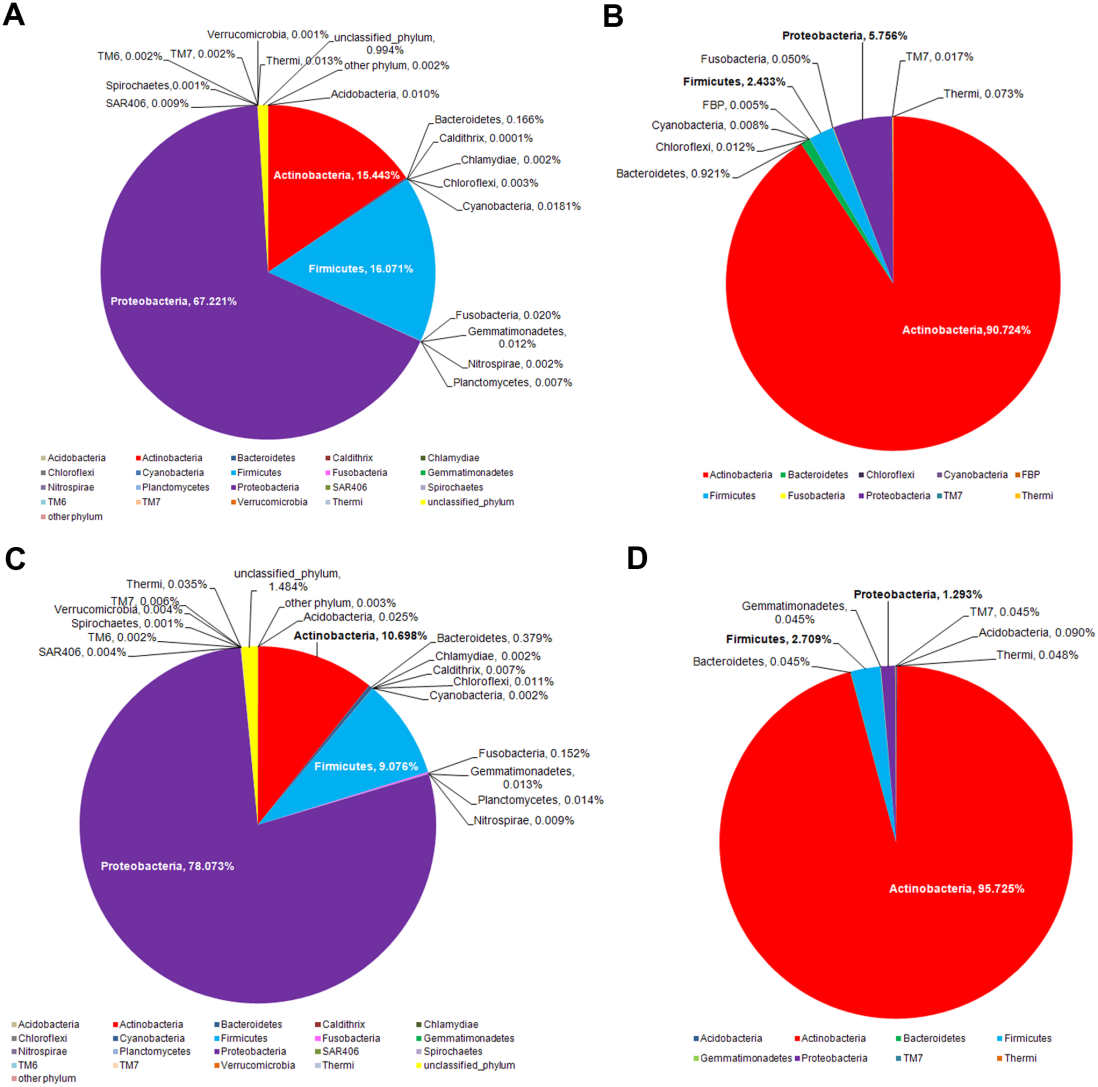
Age-matched comparison of the bacterial phylum diversity between Thai (this study) and US (data from Costello et al., 2009) cohorts of (A and B) middle and (C and D) elderly ages.

The intervariation in the facial skin bacteria between age-matched Thai and US biogeographies were then depicted. A significantly different microbial diversity was observed at the phylum level, both for the middle-aged and elderly age groups (Fig. 5). While the major phyla were all present in both biogeographies, members of Proteobacteria were more prevalent in Thai males (Fig. 5),which is the same pattern that differentiated Thai *elderly.hea* and *middle.hea* from Thai *teenage.hea* (Fig. 4A). In contrast, the US males had relatively abundant members of Actinobacteria, and these were even more dominant in the elderly (Fig. 5). Note that comparison of the teenagers was not performed since the age-matched data were not found.

### Diversity correlation by skin characteristics

Metastats was also performed to analyze the microbial community compositions with the clinical features in Table 1. The statistics demonstrated significant Spearman’s correlation (*p* < 0.05) of the microbiota patterns with the wrinkles, pores and mottled hyperpigmentation for the elderly, and flushing, oil zone and acne for the teenage. The directions were similar to those driven by Rhizobiales (*Sphingomonas* and *Stenotrophomonas*) for the *elderly.hea* and middle.hea.foreheads1, and by *Propionibacterium* and *Staphylococcus* for the teenage groups (Figs. 4B and 4C). Note that skin roughness did not show a significant correlation (Fig. 4C; *p* = 0.13). Adequate drinking water intake and hours of sleeps per day also exhibited significant correlation (Fig. 4C).

### Comparison of metabolic potentials

**Figure 6:**
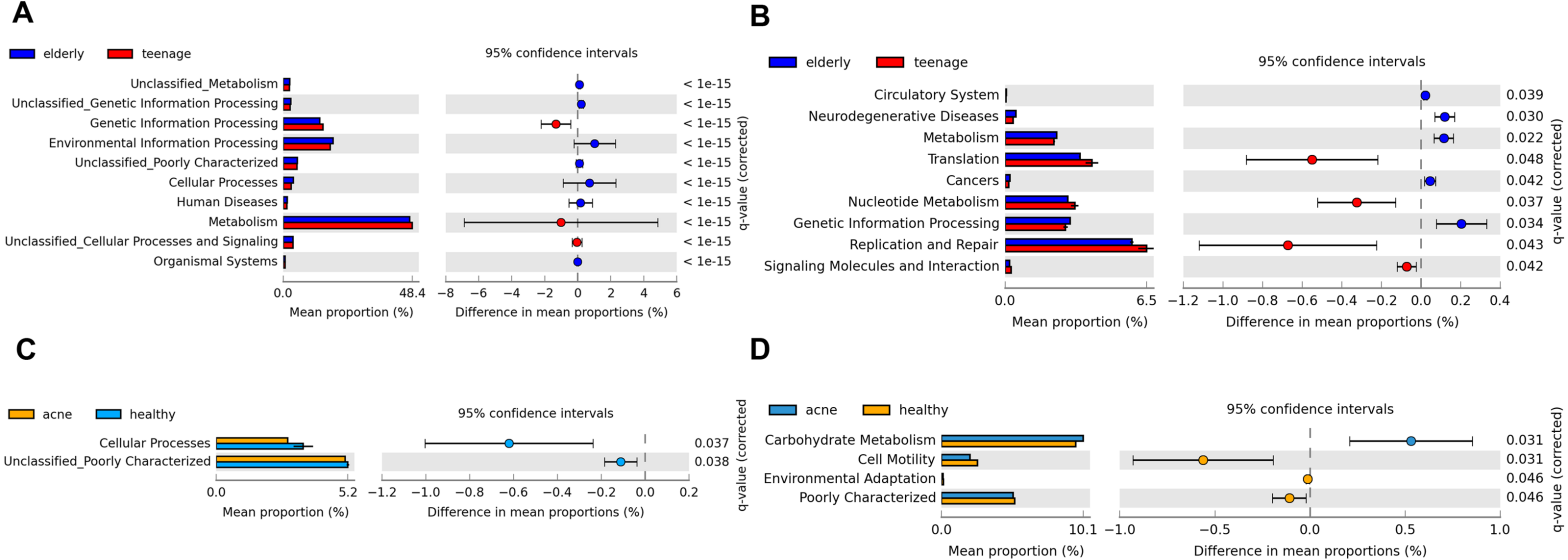
Potential metabolic functions in KEGG pathways (levels 1 and 2) between (A and B) the *elderly.hea* and *teenage.hea* and between (C and D) *teenage.acn* and *teenage.hea*.

As each bacteria species possess specific KEGG pathways, the functional potentials of the bacteria community could be predicted (Langille et al., 2013). Significant differences in the predicted KEGG functional pathways were found primarily between *elderly.hea* vs. *teenage.hea*, followed by between *teenage.acn* vs. *teenage.hea* (Fig. 6). Examples of different microbial functional pathways in aging were metabolism, genetic information processing, and human diseases (Figs. 6A and 6B). With respect to the acne, examples of different KEGG metabolic pathways were cell motility, environmental adaptation and carbohydrate metabolism. While several of the depicted metabolisms were lower for the *teenage.acn* than the *teenage.hea*, the carbohydrate metabolism was higher (Figs. 6C and 6D).

## Discussion

Metagenomic approaches, such as B-RISA and NGS, allow the in-depth evaluation and database generation of the microbial diversity, without the limitation of cultivability. The high Good’s coverage indices indicated the sufficiency of the NGS (16S rRNA gene) sequencing reads that covered over 98% of the diversity at the genus level (Table 3) (Kennedy et al., 2014). The small amount of unclassified bacterial phylum, less than 1.6% in average (Fig. 1), was consistent with previous studies. As unclassified phyla are often denoted as derived from environmental exposure, the data inferred no significant contamination from environmental exposure or sequencing error (Salter et al., 2014). Our studies, therefore, for the first time, revealed the skin bacteria of Thai males; the data by B-RISA and 16S rDNA profiles elucidated the community difference between skin ages and types.

The B-RISA finding was consistent with our NGS data, and revealed a correlation between the cheek and forehead subgroups, whereas a significant variation among the skin ages and types (Table 2 and Fig. 2). The higher bacterial nucleic acid yield (Table 2), community alpha diversity (Table 3) and number of GLOTUs (Fig. 3) in the elderly might support a previous report on increased microbial frequency in dry skin areas (Grice et al., 2009). The clinical records confirmed that the *elderly.hea* had the relatively lowest hydration and sebaceous levels (Table 1: average oil zone of 0.00). Skin characteristic analysis using Metastats detected the oil zone and acne as major influences to the bacterial community on the young adult skin (Fig. 4C).

Detecting representative bacterial species among the skin groups using Metastats indicated Rhizobiales and *Sphingomonas*, and the wrinkles and pore skin features, within the *elderly.hea* (Figs. 4B and 4C). On the other hand, the *teenage.acn*, and the middle.hea.foreheads2 that was clustered with the *teenage.acn*, shared the signatures of *P. acnes* and *S. epidermidis*. Additionally, sequencing the 16S rRNA V3–V4 region allowed for hierarchical taxonomic classification to the species level of bacteria (Somboonna et al., 2014; Mukherjee et al., 2016). The *teenage.acn* also had prevalent *S. aureus*. These bacterial species are associated with acnes and other skin diseases (Krutmann, 2009; Roudsari et al., 2015). However, *P. acnes* normally reside in the follicles around sebaceous glands, for which the special sampling method of a deep cleansing pore strip is required to sample (Barnard et al., 2016). Thus, the prevalence of *P. acnes* found in this study merely represents the general abundance on the superficial cheek and forehead areas. Nevertheless, superficial swabs used to sample the skin in this study are widely accepted and therefore supported our current research, exploring the bacterial diversity, while also providing insight about our future goal of helping to resolve the healthy bacteria balance of facial skin (Ling et al., 2013; Barnard et al., 2016; Mukherjee et al., 2016).

The microbial metabolic potentials were different between the elderly and young adult skin, and between the healthy and acne young adult skin. Compared with the elderly, the KEGG functions involved in diseases were less prevalent but the cell replication and repair was more prevalent in the normal young adults (Fig. 6). Several unclassified KEGG pathways (level 1) and functional groups (level 2) reflect the limited KEGG database, and hence limit the interpretation of the metabolic potentials by this method.

Ying et al. (2015) studied age (young adult vs. adult vs. elderly) and geography (urban vs. rural) factors associated bacterial communities on seven skin sites and 71 healthy volunteers. While body sites showed variance in the bacterial community structures, the communities also significantly differed by age and geographic factors. The database even derived the bacterial markers and allowed an opportunity to use microbiota data to predict whether an individual is a rural or urban resident (Ying et al., 2015). Skin microbiota changes over lifetime, not only by body sites and geography (Chu et al., 2017), but also the inflammatory states which are directly and indirectly affected by ages, diets, lifestyle, clinical problems, and etc. (Zapata & Quagliarello, 2015). Following the “gut-brain-skin theory,” the diverse gut microbiota in differing age groups support the diversity of the skin microbiota, consistent with the US male cohort that were found different skin bacterial communities among ages (Costello et al., 2009), and our recent findings in Thai females that found age is a factor that brings the bacterial community relationship factor closer, i.e., normal and acne teenage skin vs. normal elderly skin (Somboonna et al., 2017). Further, we re-subsampled the data at the sequencing depth of 8,000 per subgroup to obtain two replicate datasets. Analyzing these data for relative community structure and composition relatedness (Figs. S3A and S3B) and representing GLOTUs (Figs. S4B and S4E) and clinical features (skin, diet and lifestyle) (Figs. S4C and S4F) exhibited consistent findings. Together, these supported the bacterial community variation by age and relevant skin features.

For healthy eating diet, we did not detect any significant correlation, which might correspond to general healthy eating diets among volunteers (Table 1: 80%–90%). The healthy eating pyramid defines the appropriate types and proportions of daily food consumption (e.g., food that is rich in vegetable fibers, fruit antioxidants, vegetable and fish proteins, and low glycemic load). The lack of variation in healthy eating pyramid factor across our study groups might have obfuscated the Spearman’s correlation findings, as we did not find the healthy eating pyramid related with any microbiota structures (Fig. 4C and Figs. S4C and S4F). To investigate the diet factor to the skin microbiota, more detail and variations of eating habits, such as glycemic load, should be obtained and analyzed (Wu et al., 2011; Lopez-Legarrea et al., 2014). To date, oral and topical probiotics have been proposed a healthy skin therapy (Isawa et al., 2008; Kimoto-Nira et al., 2014; Huang & Tang, 2015; Kober & Bowe, 2015); our ongoing studies include tracking the effect of oral and topical probiotics on skin microbiota.

Different facial skin bacterial structures between Thai and previously reported US males (Costello et al., 2009) is in accord with previous research that suggested the skin bacteria vary across ethnicities and geographies; diet, genetic and environment factors are possible causes of this difference (HMP, 2012; Zhou et al., 2013). Here, extremophiles, such as Firmicutes, were more enriched on the Thai skin compared to the US, whereas Actinobacteria were more prevalent on the US skin (Fig. 5). This difference might potentially be involved in environmental adaptation. In support, common bacteria on dry skin were previously reported to include Proteobacteria, Firmicutes and Bacteroidetes (Grice & Segre, 2011; Staudinger, Pipal & Redl, 2011). Of additional note are the variation of V3–V4 vs. V2 sequences annotation, and that we sampled skin at ∼8 h after face wash, which more hours allowed for natural bacterial flora establishment (Costello et al., 2009).

## Conclusions

As sebum and hydration levels represent two significant predictors to the nature and diversity of the skin, this study firstly surveyed the facial skin microbiota of Thai males that differ in age and acne skin groups. In addition, the facial skin microbiome of elderly individuals has rarely been reported. Using young adults as the reference point, this study demonstrated that the facial skin bacteria population structure continued to change from young to middle age to elderly adulthood, in association with specific bacterial flora and clinical skin features. The predicted KEGG metabolic profiles of the microbiota supported the functional diversity among the different skin types. Comparison between Thai and US biogeographies showed the bacterial community differences that could be related with specific skin characteristics. Together, our findings on bacterial community structure characterizing Thai male facial skin represents the first preliminary findings to be used in other comparative studies in order to gain insights into healthy facial skin microbiota, with possible correlation with biogeography, gender, and skin types.

##  Supplemental Information

10.7717/peerj.4084/supp-1 Table S1 Primers in the B-RISA and 16S rDNA PCR amplifications Italic represents barcode sequence.

10.7717/peerj.4084/supp-2 Figure S1 Variance analysis by Boxplot of the 16S rDNA MiSeq data between 2 *middle.hea* foreheads subgroups, 2 *teenage.acn* cheeks subgroups, 2 *teenage.acn* foreheads subgroups, and 2 *teenage.hea* foreheads subgroups, respectively

10.7717/peerj.4084/supp-3 Figure S2A Estimate GLOTU richness by rarefaction curves of cheek datasets

10.7717/peerj.4084/supp-4 Figure S2B Estimate GLOTU richness by rarefaction curves of forehead datasets

10.7717/peerj.4084/supp-5 Figure S3A Diversity and relative abundances of bacterial community compositions (as GLOTU) of the first (A) and second (B) subsampling datasets (Left) dendrogram computed with Morisita-Horn dissimilarity indices: teenage (red), middle-aged (green) and elderly (navy). Bacterial genera of <0.05% of relative abundance are not displayed.

10.7717/peerj.4084/supp-6 Figure S3B

10.7717/peerj.4084/supp-7 Figure S4A Non-metric multidimensional scaling based on Morisita-Horn dissimilarity indices and Metastats analyses of the first (A–C) and second (D–F) subsampling datasets (A and D) without, and with Metastats analyses for representative (B and E) GLOTUs and (C and F) clinical features (skin, diet and lifestyle). The vector length indicates the strength of the association. The direction infers the direction of the effect. For Metastats analyses, arrow with red font indicates the detection with significant statistics (*p* < 0.05), and arrow with the smaller size and in gray font (i.e. *Corynebacterium*, and roughness) indicates the detection with non-significant statistics (*p* > 0.05).

10.7717/peerj.4084/supp-8 Figure S4B

10.7717/peerj.4084/supp-9 Figure S4C

10.7717/peerj.4084/supp-10 Figure S4D

10.7717/peerj.4084/supp-11 Figure S4E

10.7717/peerj.4084/supp-12 Figure S4F

## Additional Information and Declarations

### Data Availability

The following information was supplied regarding data availability: All nucleic acid sequences in this study are accessible via the NCBI Sequence Read Archive (SRA) database (accession number SRR3656927).
