# Supplementary material for: Diversity of bacterial communities on the facial skin of different age-group Thai males
Source: PeerJ. 2017 Nov 21;5:e4084. doi: 10.7717/peerj.4084 (PMC5701550; doi:10.7717/peerj.4084)
Supplement: Table S1 — Italic represents barcode sequence. [file peerj-05-4084-s001.docx]

**Supplemental Table 1**

| **Sample names** | **Forward primers (5′-3′)** | **Reverse primers (5′-3′)** |
| --- | --- | --- |
| B-RISA | GYACACACCGCCCGT | GGGTTBCCCCATTCRG |
| elderly.hea.cheeks | *ATGTAGAC*ACTCCTACGGGAGGCAGCAG | *ATGTAGAC*CTACCAGGGTATCTAATC |
| elderly.hea.foreheads | *TCTCGCAC*ACTCCTACGGGAGGCAGCAG | *TCTCGCAC*CTACCAGGGTATCTAATC |
| middle.hea.foreheads1 | *ACTGTGTC*ACTCCTACGGGAGGCAGCAG | *ACTGTGTC*CTACCAGGGTATCTAATC |
| middle.hea.foreheads2 | *ATGTGAGC*ACTCCTACGGGAGGCAGCAG | *ATGTGAGC*CTACCAGGGTATCTAATC |
| teenage.hea.cheeks | *AGTATCTG*ACTCCTACGGGAGGCAGCAG | *AGTATCTG*CTACCAGGGTATCTAATC |
| teenage.hea.foreheads1 | *TCGTATAG*ACTCCTACGGGAGGCAGCAG | *TCGTATAG*CTACCAGGGTATCTAATC |
| teenage.hea.foreheads2 | *TCGTATAG*ACTCCTACGGGAGGCAGCAG | *TCGTATAG*CTACCAGGGTATCTAATC |
| teenage.acn.cheeks1 | *ATCGCGCG*ACTCCTACGGGAGGCAGCAG | *ATCGCGCG*CTACCAGGGTATCTAATC |
| teenage.acn.cheeks2 | *TCATGCGC*ACTCCTACGGGAGGCAGCAG | *TCATGCGC*CTACCAGGGTATCTAATC |
| teenage.acn.foreheads1 | *TCATAGAC*ACTCCTACGGGAGGCAGCAG | *TCATAGAC*CTACCAGGGTATCTAATC |
| teenage.acn.foreheads2 | *AGACATAG*ACTCCTACGGGAGGCAGCAG | *AGACATAG*CTACCAGGGTATCTAATC |
