# Supplementary figures and images for: Diversity of bacterial communities on the facial skin of different age-group Thai males
Source: PeerJ. 2017 Nov 21;5:e4084. doi: 10.7717/peerj.4084 (PMC5701550; doi:10.7717/peerj.4084)

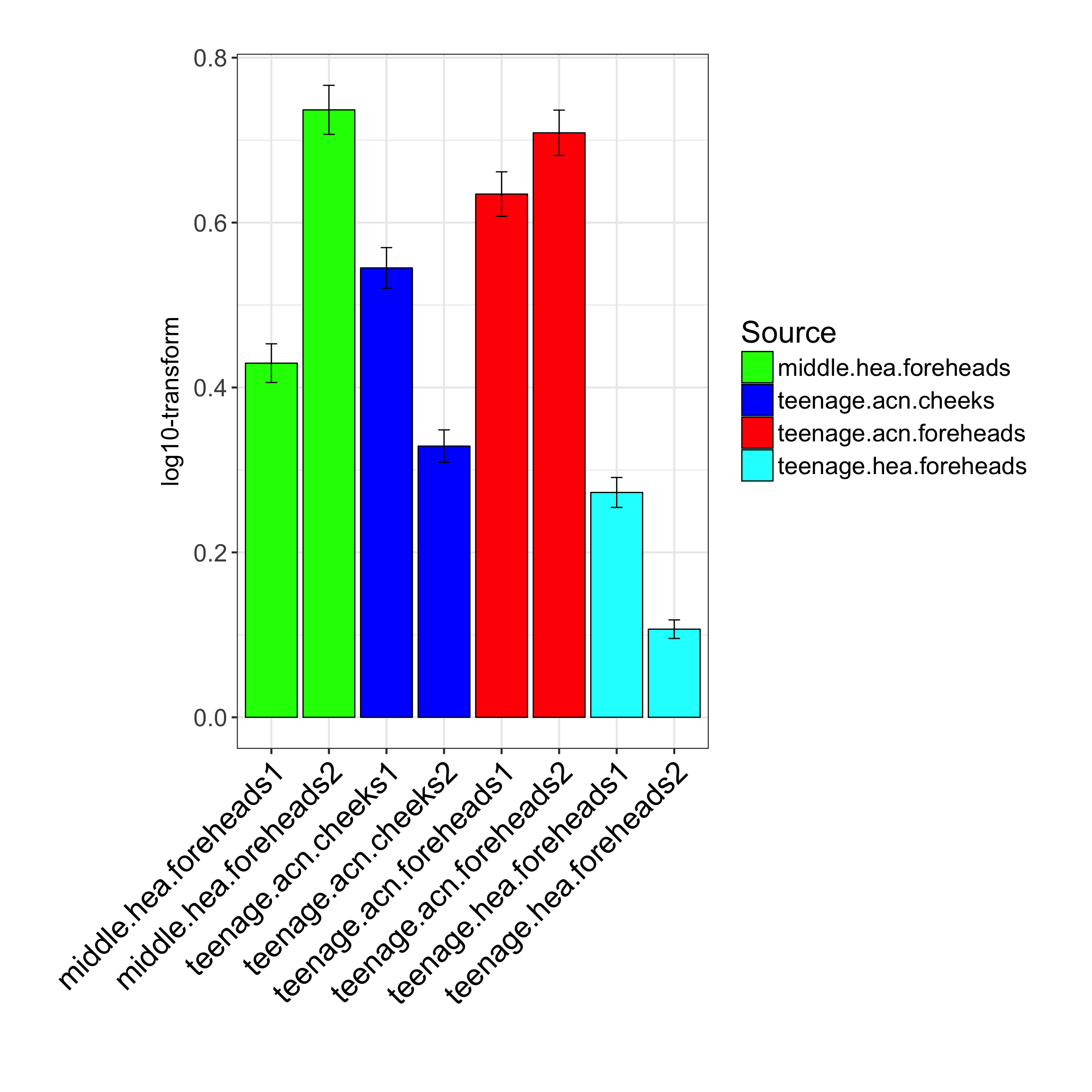

Supplement: Figure S1 [file peerj-05-4084-s002.png]

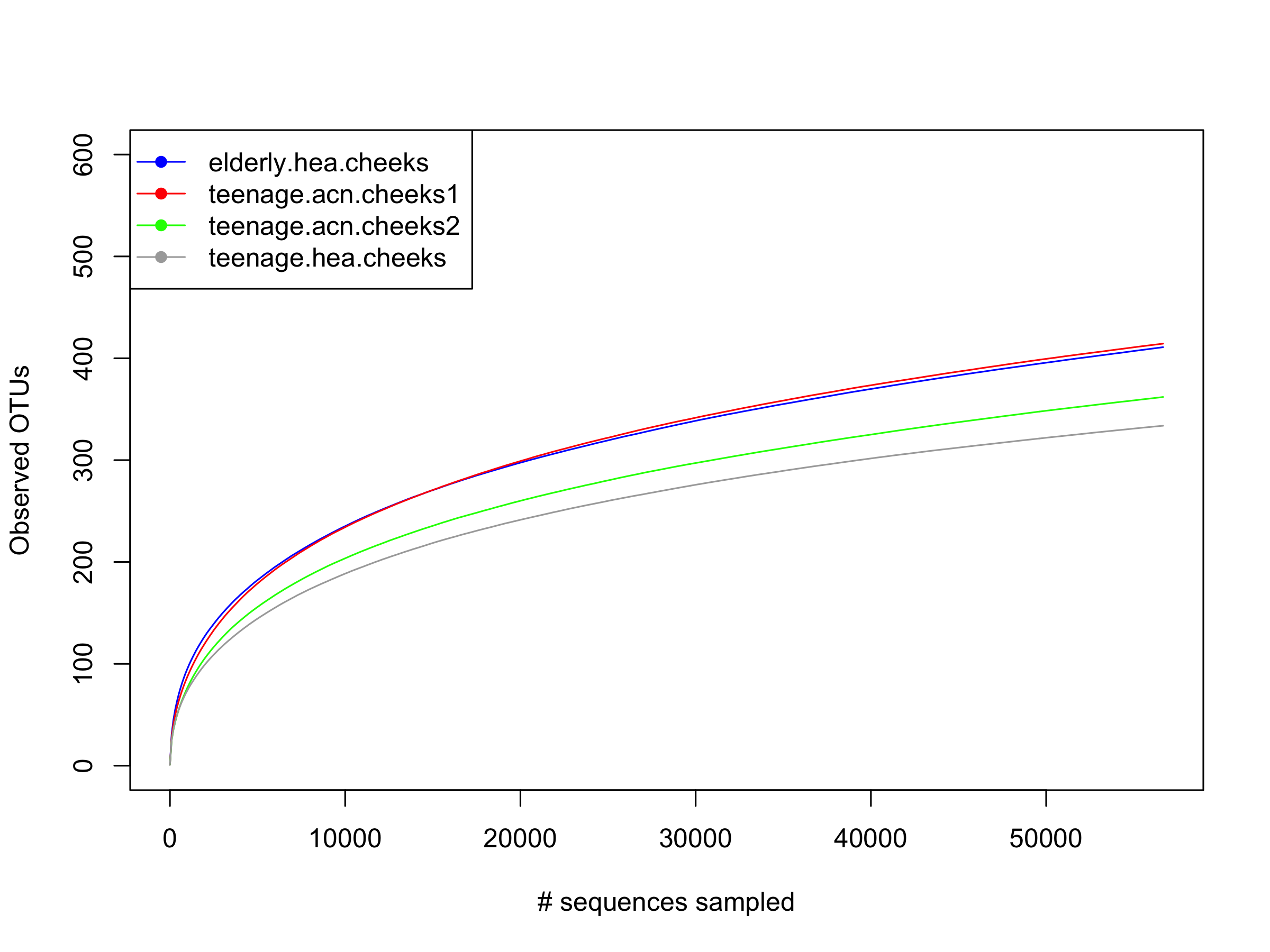

Supplement: Figure S2A [file peerj-05-4084-s003.png]

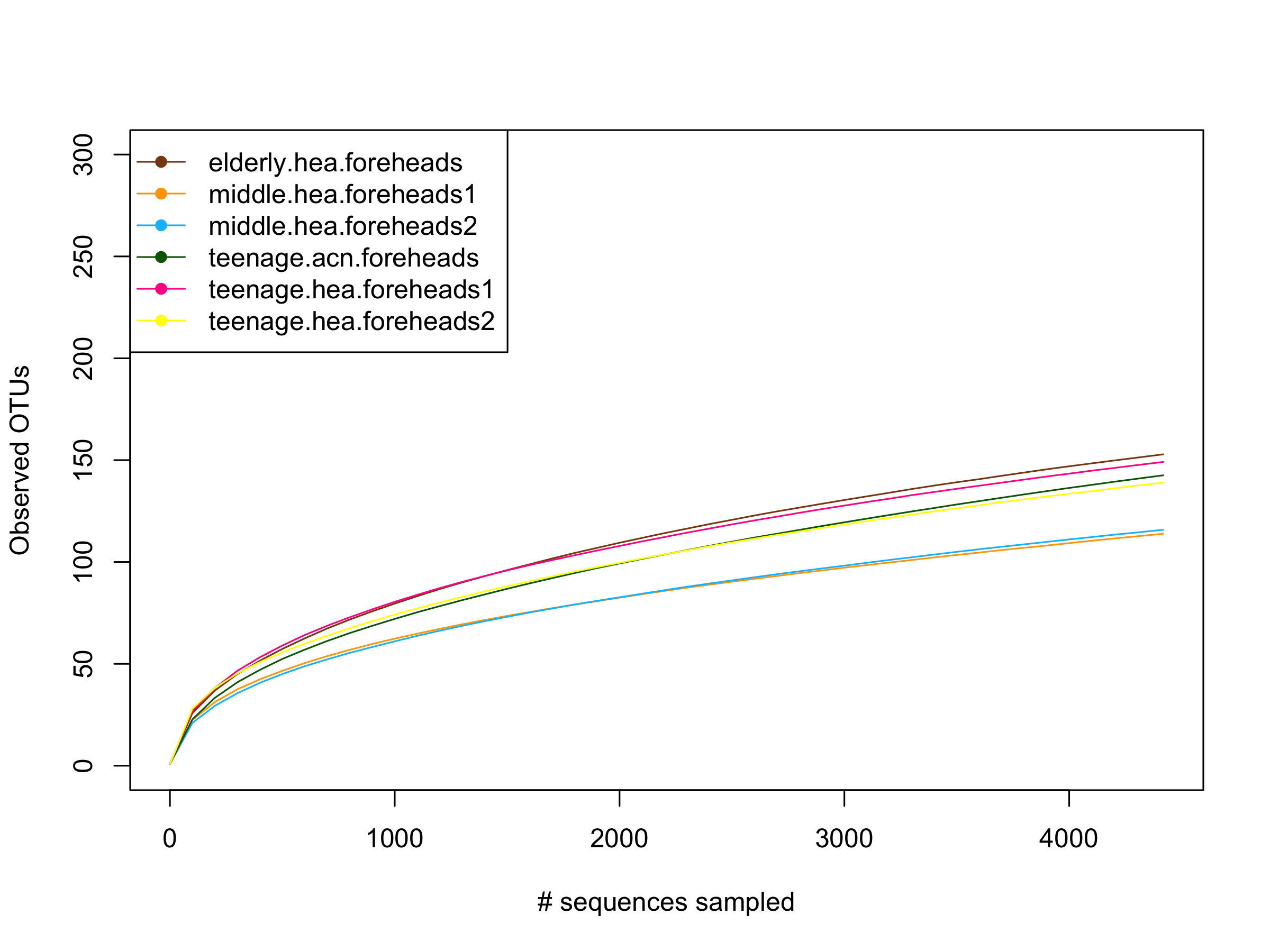

Supplement: Figure S2B [file peerj-05-4084-s004.png]

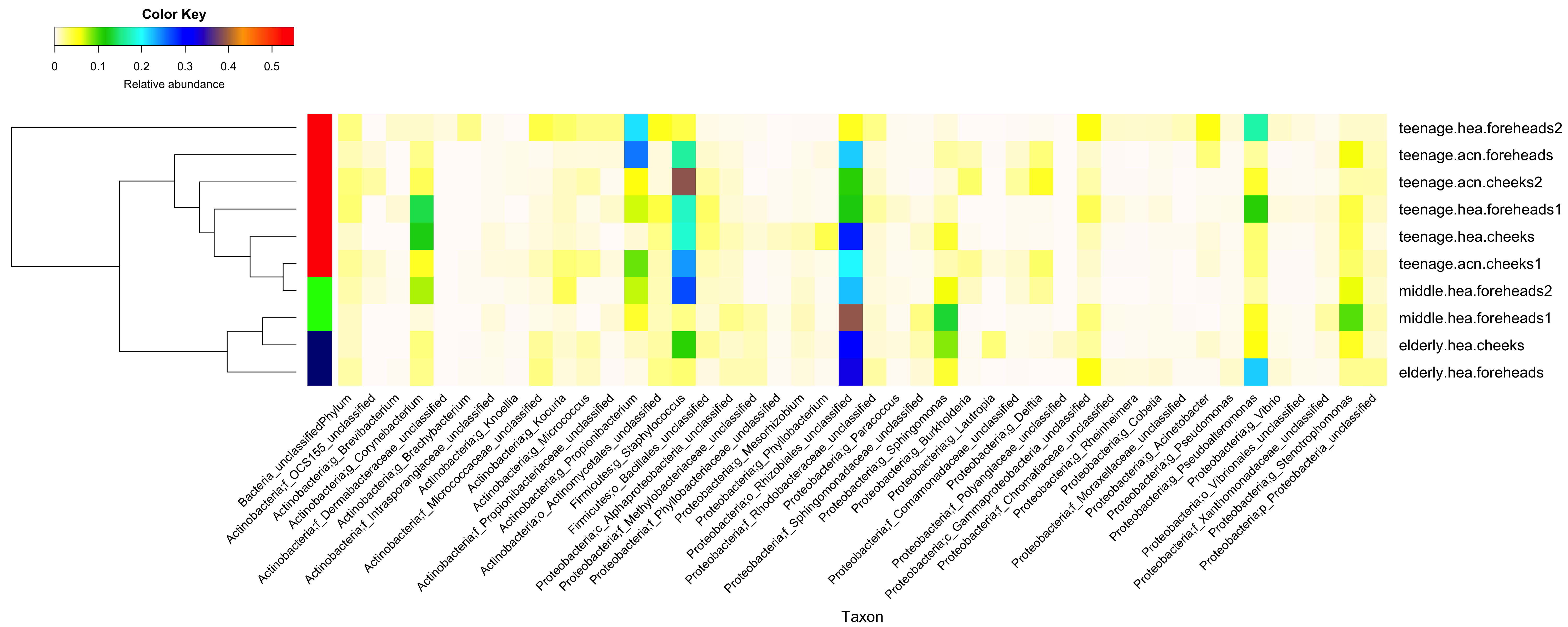

Supplement: Figure S3A — (Left) dendrogram computed with Morisita-Horn dissimilarity indices: teenage (red), middle-aged (green) and elderly (navy). Bacterial genera of <0.05% of relative abundance are not displayed. [file peerj-05-4084-s005.png]

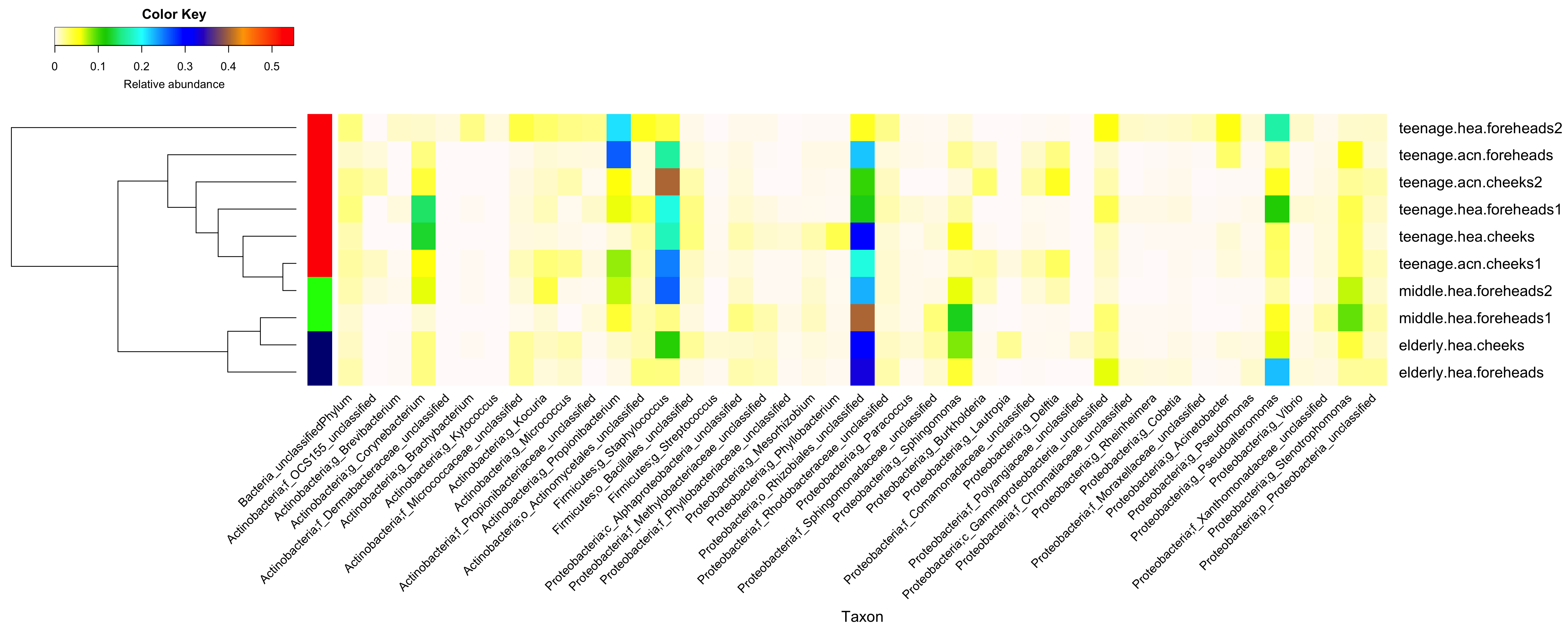

Supplement: Figure S3B [file peerj-05-4084-s006.png]

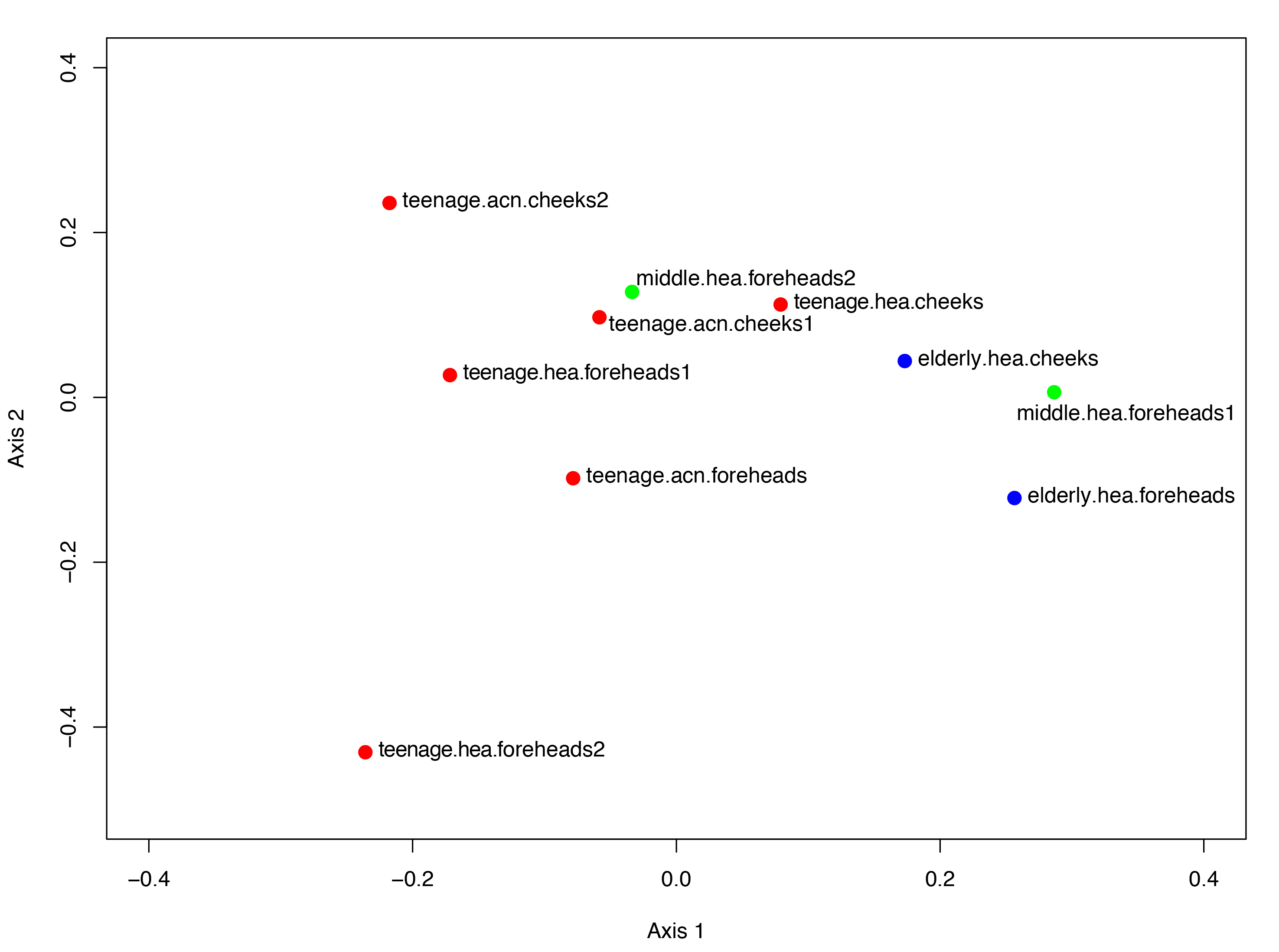

Supplement: Figure S4A — (A and D) without, and with Metastats analyses for representative (B and E) GLOTUs and (C and F) clinical features (skin, diet and lifestyle). The vector length indicates the strength of the association. The direction infers the direction of the effect. For Metastats analyses, arrow with red font indicates the detection with significant statistics (p < 0.05), and arrow with the smaller size and in gray font (i.e. Corynebacterium, and roughness) indicates the detection with non-significant statistics (p > 0.05). [file peerj-05-4084-s007.png]

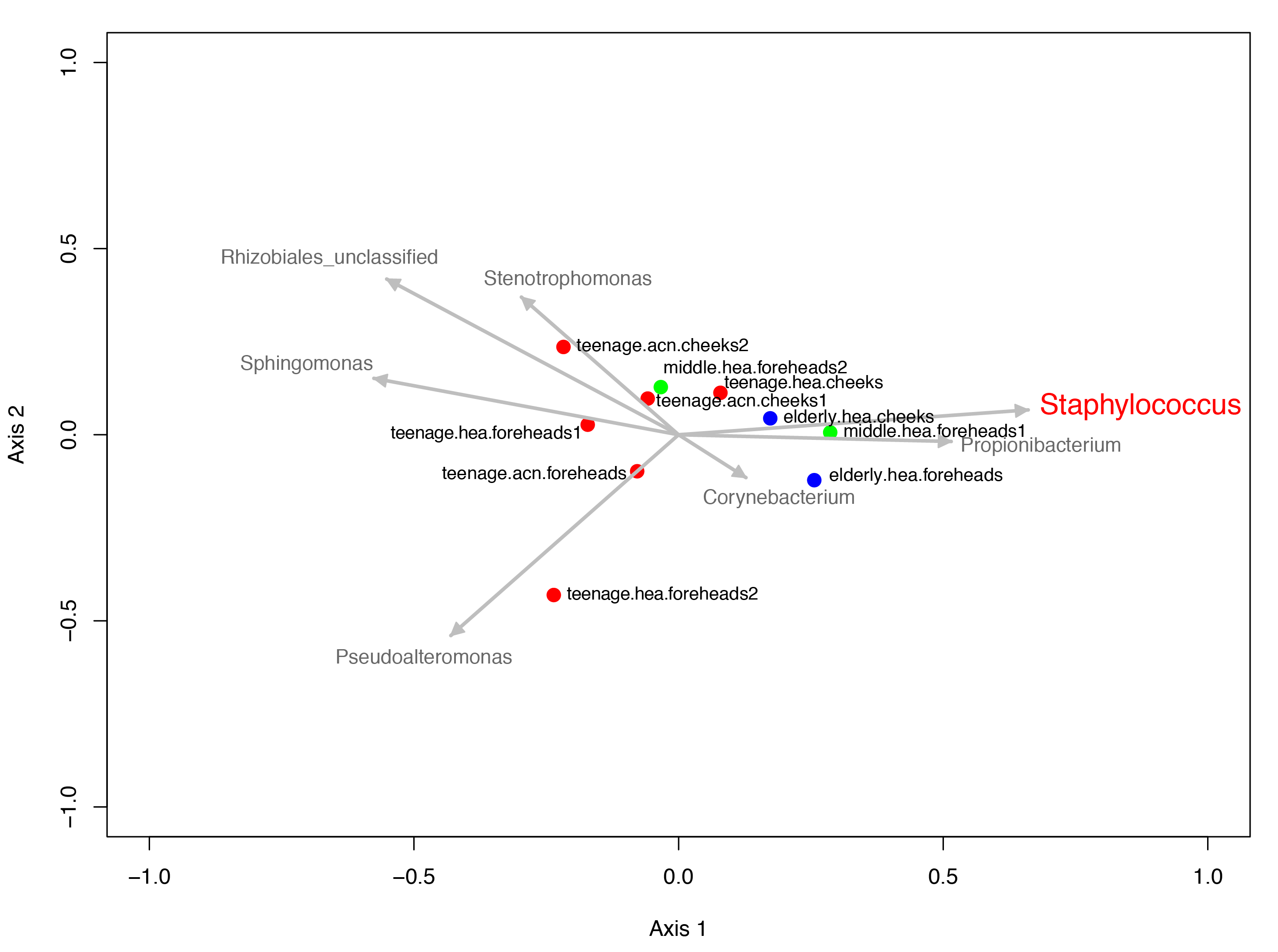

Supplement: Figure S4B [file peerj-05-4084-s008.png]

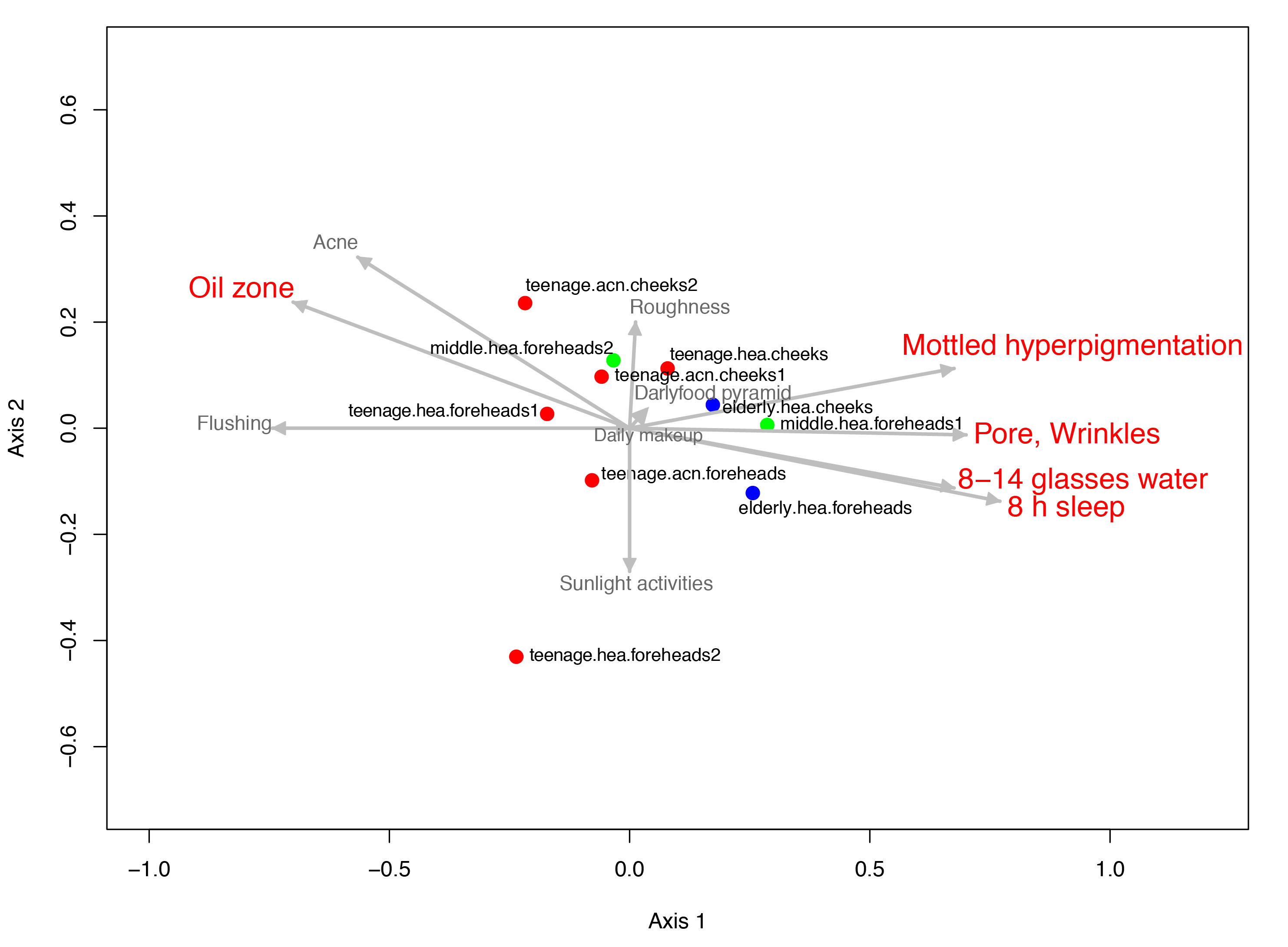

Supplement: Figure S4C [file peerj-05-4084-s009.png]

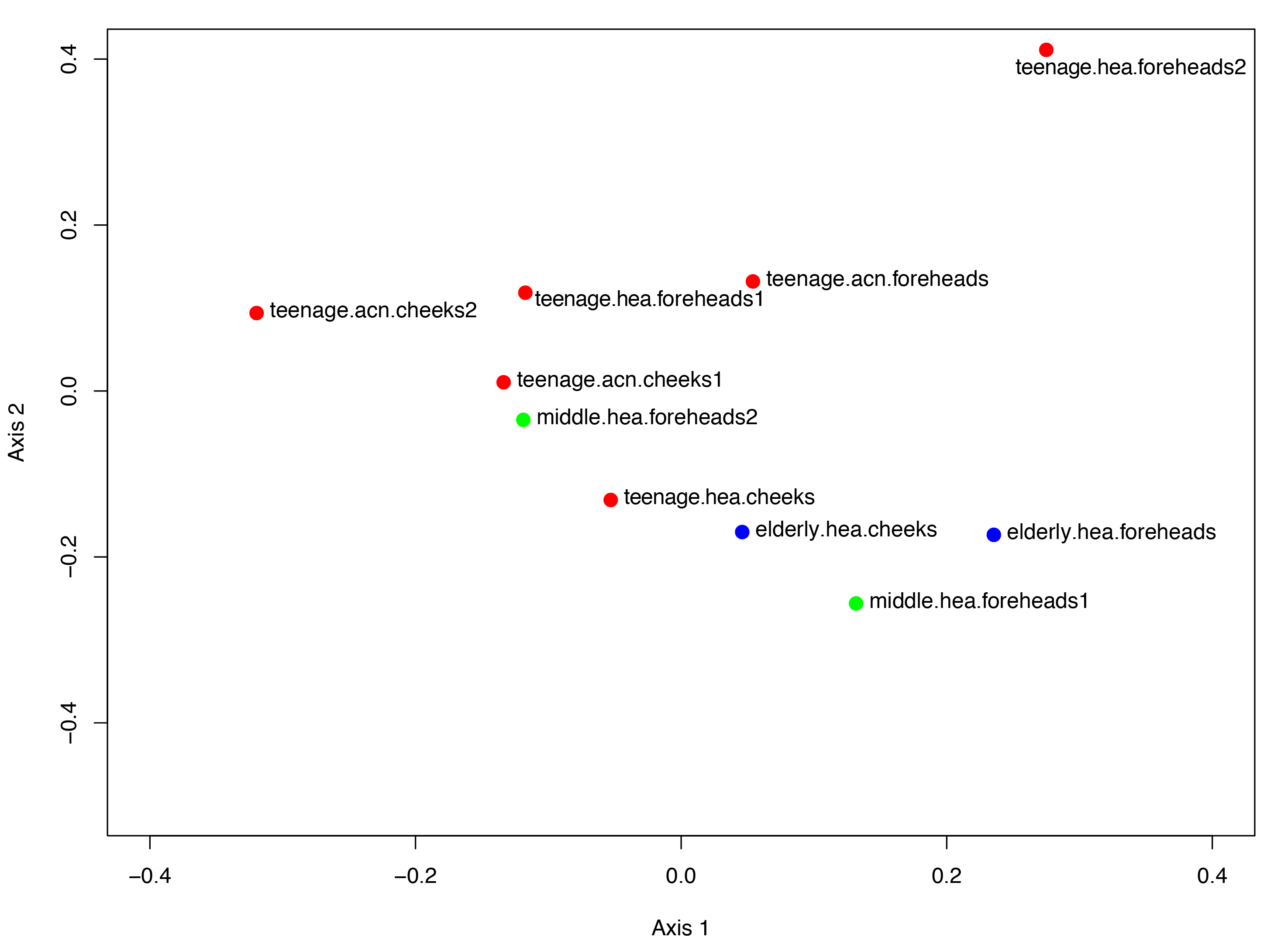

Supplement: Figure S4D [file peerj-05-4084-s010.png]

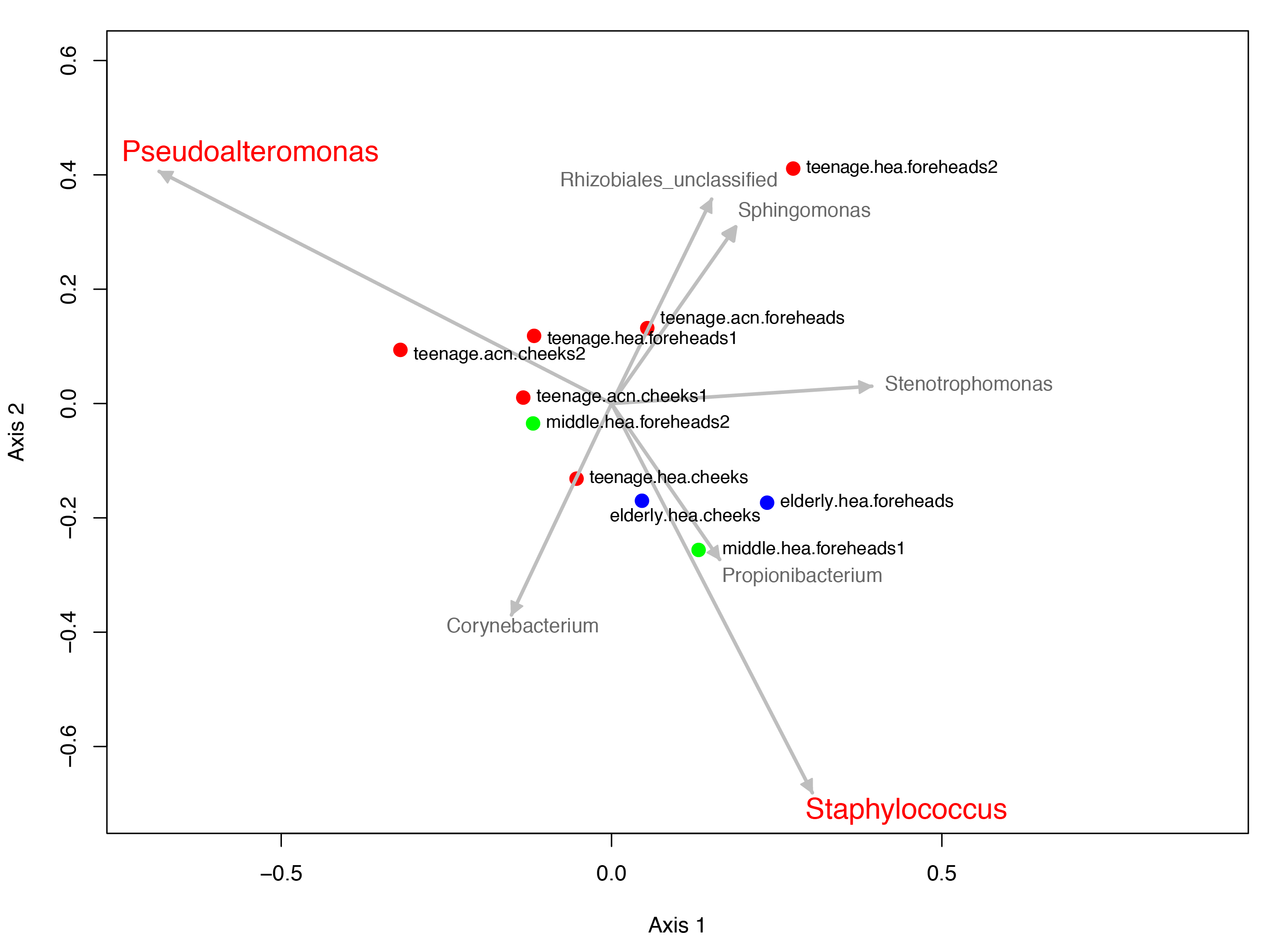

Supplement: Figure S4E [file peerj-05-4084-s011.png]

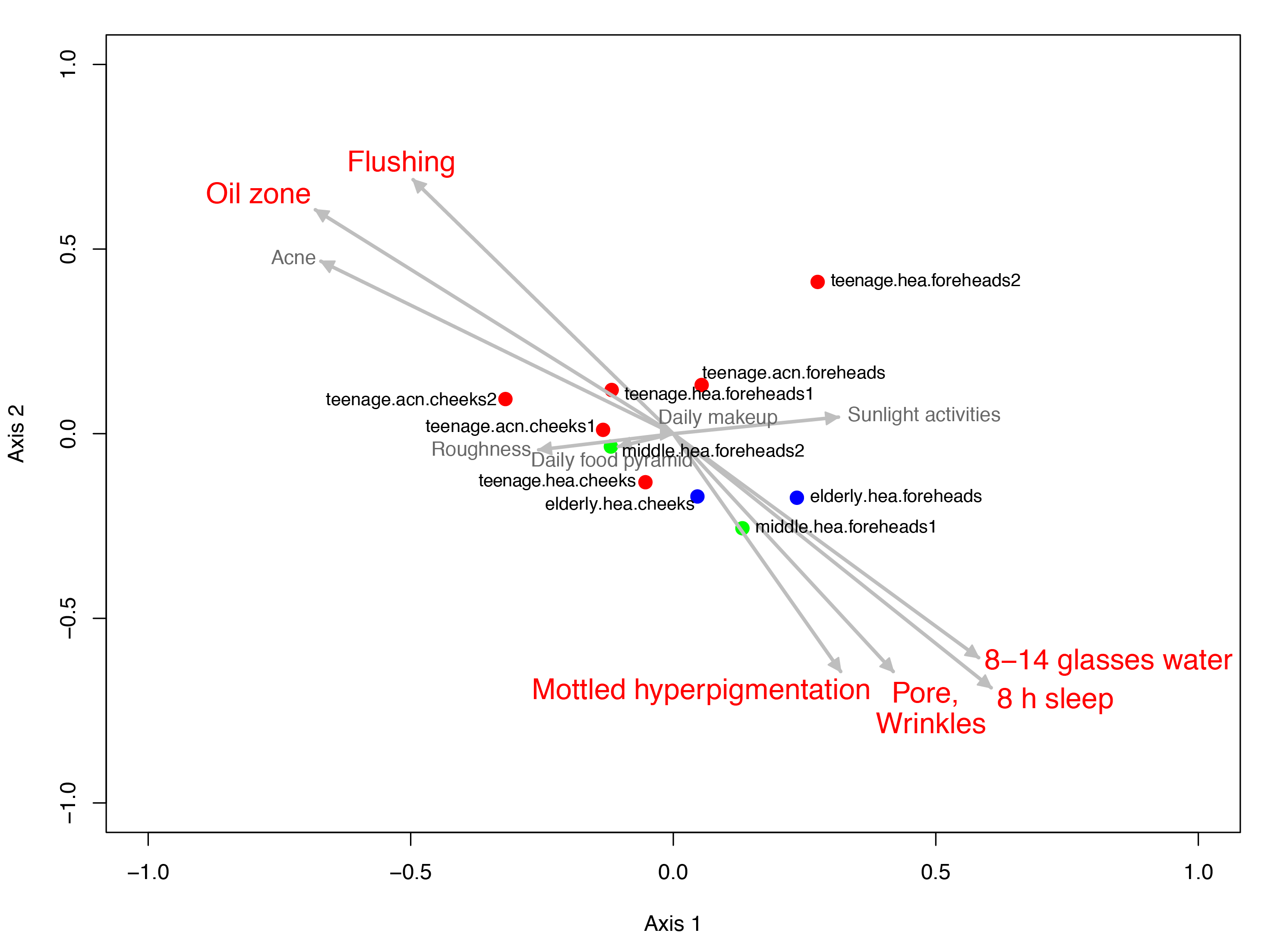

Supplement: Figure S4F [file peerj-05-4084-s012.png]
